# Mechanosensitive Piezo1 channels mediate renal fibrosis

**DOI:** 10.1172/jci.insight.152330

**Published:** 2022-04-08

**Authors:** Xiaoduo Zhao, Yonglun Kong, Baien Liang, Jinhai Xu, Yu Lin, Nan Zhou, Jing Li, Bin Jiang, Jianding Cheng, Chunling Li, Weidong Wang

**Affiliations:** 1 Department of Pathophysiology,; 2 Institute of Hypertension, and; 3 Department of Physiology, Zhongshan School of Medicine, Sun Yat-sen University, Guangzhou, China.; 4 Department of Pathology, Zhujiang Hospital, Southern Medical University, Guangzhou, China.; 5 Department of Forensic Medicine, Zhongshan School of Medicine, Sun Yat-sen University, Guangzhou, China.; 6 The First Affiliated Hospital, Guangzhou University of Chinese Medicine, Guangzhou, China.; 7 Department of Nephrology, The Seventh Affiliated Hospital, Sun Yat-sen University, Shenzhen, China.

**Keywords:** Nephrology, Chronic kidney disease, Fibrosis, Ion channels

## Abstract

Kidney fibrosis is the final common pathway of progressive kidney diseases, the underlying mechanisms of which are not fully understood. The purpose of the current study is to investigate a role of Piezo1, a mechanosensitive nonselective cation channel, in kidney fibrosis. In human fibrotic kidneys, Piezo1 protein expression was markedly upregulated. The abundance of Piezo1 protein in kidneys of mice with unilateral ureter obstruction (UUO) or with folic acid treatment was significantly increased. Inhibition of Piezo1 with nonspecific inhibitor GsMTx4 markedly ameliorated UUO- or folic acid–induced kidney fibrosis. Mechanical stretch, compression, or stiffness induced Piezo1 activation and profibrotic responses in human HK2 cells and primary cultured mouse proximal tubular cells (mPTCs), which were greatly prevented by inhibition or silence of Piezo1. TGF-β_1_ induced increased Piezo1 expression and profibrotic phenotypic alterations in HK2 cells and mPTCs, which were again markedly prevented by inhibition of Piezo1. Activation of Piezo1 by Yoda1, a Piezo1 agonist, caused calcium influx and profibrotic responses in HK2 cells and induced calcium-dependent protease calpain2 activation, followed by adhesion complex protein talin1 cleavage and upregulation of integrin β_1_. Also, Yoda1 promoted the link between ECM and integrin β_1_. In conclusion, Piezo1 is involved in the progression of kidney fibrosis and profibrotic alterations in renal proximal tubular cells, likely through activating calcium/calpain2/integrin β_1_ pathway.

## Introduction

Kidney fibrosis is the final common outcome of chronic kidney diseases, leading to the end stage of kidney diseases (1). The pathophysiological events during renal tubulointerstitial fibrogenesis are complex and involve several independent and overlapping cellular and molecular signaling pathways. Renal fibrogenesis is a dynamic and converging process, in which several types of cells, mainly tubular epithelial cells and interstitial fibroblasts, play important roles in tubulointerstitial fibrosis after an insult to the renal parenchyma (2).

Kidney fibrosis is characterized by increased synthesis and inadequate degradation of extracellular matrix (ECM) in tubulointerstitial areas, which makes fibrotic regions much stiffer than normal kidney tissue (1). Tubular epithelia in the kidney are exposed to different mechanical forces, from ECM on the basal side and from fluid shear stress on the luminal side. Tubular epithelium is able to detect tiny changes in matrix stiffness and, in response, to adjust expression of genes and proteins important in cell proliferation, differentiation, and apoptosis (3, 4). After severe or repeated injuries, tubular epithelial cells may exhibit a series of characteristic alterations and acquire mesenchymal traits, leading to proinflammatory and profibrotic phenotypic changes (5, 6). An epithelial-mesenchymal transition (EMT) phenomenon was found in vitro after stimulation by transforming growth factor-β_1_ (TGF-β_1_), a well-known profibrotic factor, but multiple lineage tracing studies do not support the contribution of in vivo EMT to kidney fibrosis (7, 8). It is possible that EMT may represent tubular injuries, epithelial plasticity, or profibrotic phenotypic changes. TGF-β_1_ may directly induce tubular injuries and the production of ECM in the kidney through TGF-β_1_/Smad signaling, leading to renal fibrosis (9).

It has been known that epithelium may sense the microenvironments and transduce outside mechanical signals to intracellular chemical and electronic signals through mechanoreceptors, which include ion channels, integrins, G protein–coupled receptors, glycocalyx, and so on (10–12). Piezo1 is a newly found mechanosensitive nonselective cation channel, which extensively distributes in multiple cell types and tissues (13). Piezo1 is gated and tuned by cellular membrane bilayer tension directly (14). Physical forces, such as shear stress, compression, stretch, and osmotic stress, induce a change in membrane tension and open the Piezo1 channel to allow permeation of cations (K^+^, Na^+^, Ca^2+^, and Mg^2+^) with a slight preference for calcium (15). Piezo1 plays an important role in numerous physiological processes, such as determining vascular structure (16), urine osmoregulation (17), and blood pressure homeostasis (18). In the kidney, Piezo1 protein expression was found in the renal corpuscle, proximal and distal convoluted tubule, and cortical and medullary collecting duct (17, 19).

Sensing and responding to mechanical signals are critical for the proper functioning of the nephrons and collecting ducts. Renal epithelial cells respond to changes in tubular fluid flow, intraluminal pressure, and wall tension (20). When fibrotic lesions are present, kidney gradually stiffens, which is usually driven by the replacement of compliant cells with rigid matrix (such as collagen remodeling and the consequent activation in ECM signaling) and is further increased by cross-linking of these matrix fibrils (21). Normal stiffness of kidney cortex is about 4 kPa; stiffness of cortex may increase to 35 kPa in human kidney with fibrotic pathological changes (22, 23). A previous study showed that Piezo1 senses microenvironmental stiffness and transduces the mechanical cues into electronic and chemical signals in cells (24). Piezo1 was also shown to be involved in myofibroblast-fibroblast crosstalk in fibrosis expansion (25). Mechanical stretch–induced EMT in alveolar epithelia (26) or in cholangiocarcinoma cells has been demonstrated via Piezo1 activation (27). It is recognized that mechanical stimulus is closely associated with kidney fibrosis; whether Piezo1 as a candidate mechanosensor is involved in progression of renal fibrosis is still unknown.

Integrins are also mechanoreceptors and are transmembrane protein complexes formed by α and β subunits. The extracellular domain of integrin interacts with ECM proteins and cytoplasmic domain interacts with cytoskeleton (28). When integrin binds to ECM proteins, outside environmental mechanical signals can be transmitted into cells, activating integrin-dependent intracellular kinase signaling to regulate cellular physiological processes. On the other hand, intracellular signals, especially calcium signals, activate intracellular domain of integrins, followed by clustering of integrins and the increased affinity of the integrins to the ECM ligand. Emerging evidence has shown an association between activation of Piezo1 and integrin-related signaling (29, 30).

In the current study, we hypothesized that activation of Piezo1 in renal tubular epithelia may contribute to the development of kidney interstitial fibrosis. We demonstrated that inhibition of Piezo1 at least partially prevented unilateral ureter obstruction (UUO) or folic acid–induced renal fibrosis. In vitro study showed that stimulation of Piezo1 induced profibrotic phenotypic changes in tubular cells, likely through activating calcium/calpain/integrin signaling. Targeting the Piezo1 pathway may offer a novel therapeutic strategy for ameliorating renal fibrosis.

## Results

### Renal fibrosis is associated with increased Piezo1 protein expression.

**Figure 1:**
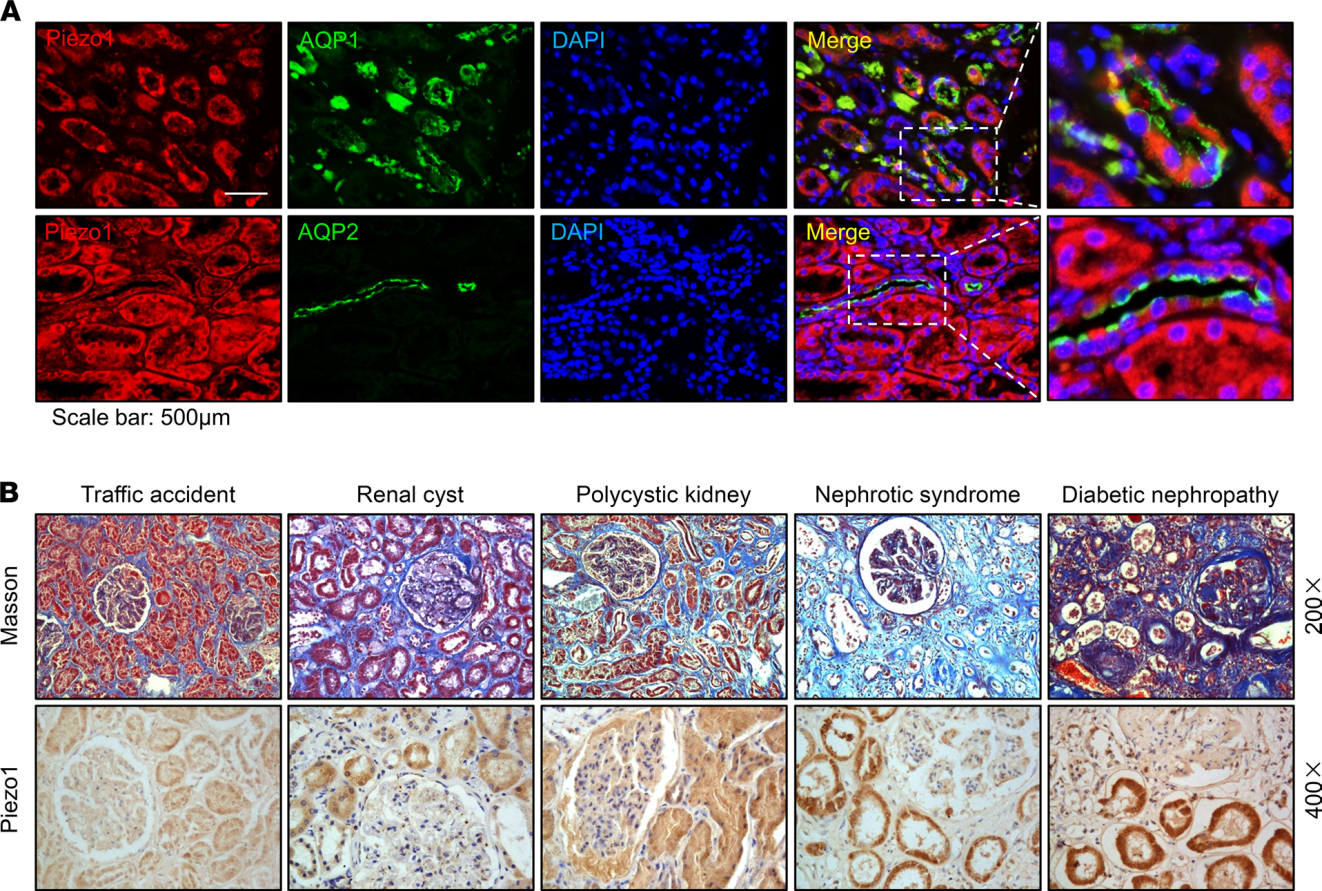
Increased expression of Piezo1 protein in fibrotic kidney diseases. (**A**) Colocalization of Piezo1 (red) and AQP1 (green) or AQP2 (green) in the kidney specimens from decedents who died of traffic accidents, detected by immunofluorescence. Scale bars: 500 μm, 217 μm (insets). (**B**) Representative photomicrographs of Masson’s staining (original magnification, 200×) and immunohistochemistry (original magnification, 400×) of Piezo1 in the kidney specimens from decedents with different kidney diseases. AQP1, aquaporin-1; AQP2, aquaporin-2.

In order to examine a potential association between expression abundance of Piezo1 protein and fibrosis, Piezo1 labeling was performed on kidney specimens obtained at autopsy of decedents. The human kidney specimens, from decedents died from traffic accidents, with minimal pathological changes were used as controls. Water channel aquaporin-1 (AQP1) is specially located in the proximal tubular cells, and AQP2 is located in the collecting duct principal cells in the kidneys. Immunofluorescence showed that Piezo1 was generally expressed in human kidney tubules, including proximal tubular cells (colocalized with AQP1) and collecting duct principal cells (colocalized with AQP2) (Figure 1A). Masson’s trichrome staining showed more fibrotic lesions in specimens obtained from decedents with renal cysts, polycystic kidney, nephrotic syndrome, or diabetic nephropathy than those from controls. Interestingly, in fibrotic kidney specimens more tubular labeling of Piezo1, particularly in proximal tubules, was found than in controls by immunohistochemistry (Figure 1B).

**Figure 2:**
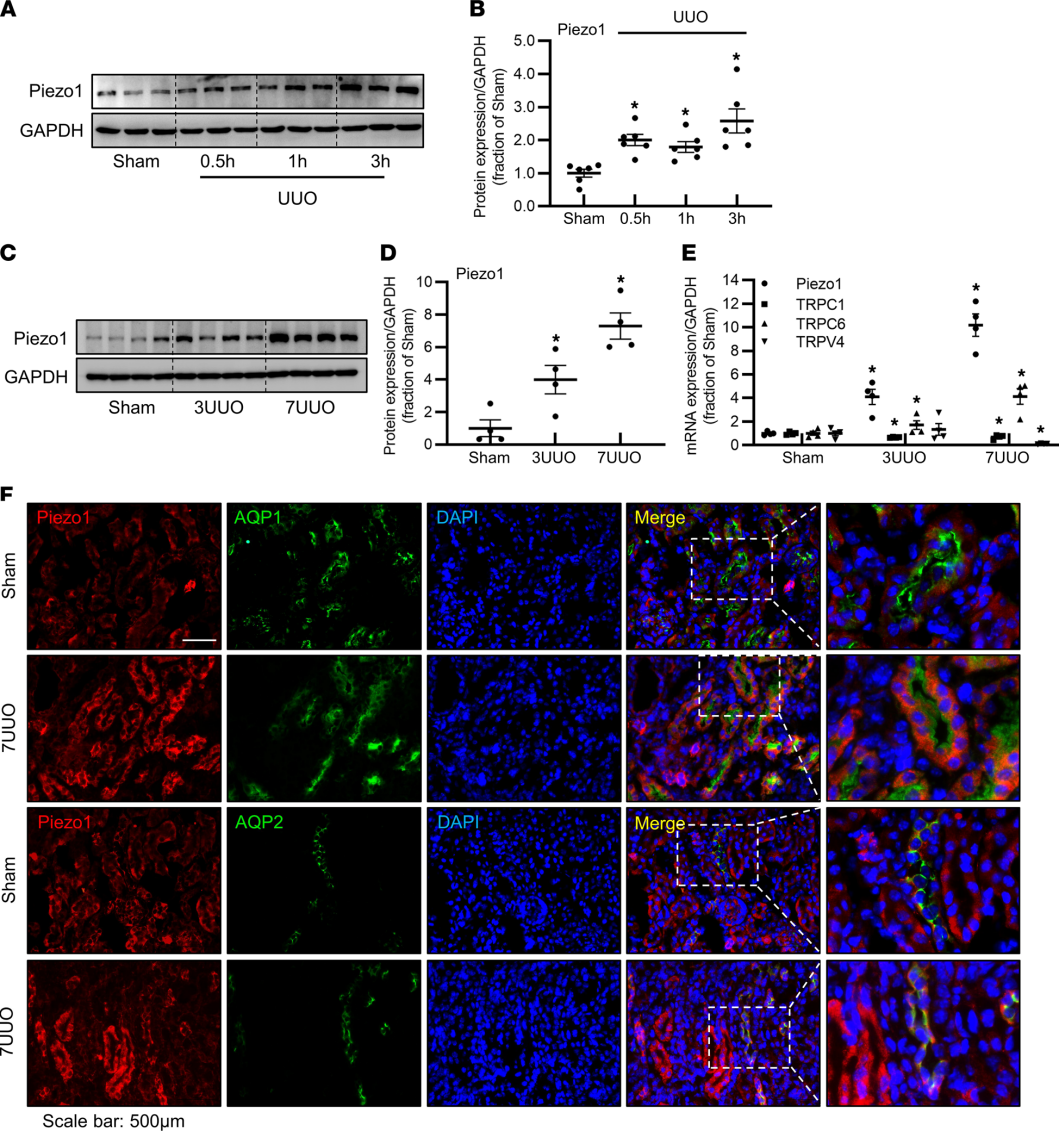
Increased expression of Piezo1 protein and mRNA in UUO mice. (**A** and **B**) Representative immunoblots and corresponding densitometry analysis of Piezo1 protein abundance in the kidney of sham and UUO mice at 30 minutes, the first hour, and the third hour after surgery. Data are shown as mean ± SEM (*n* = 6 in each group). (**C** and **D**) Representative immunoblots and corresponding densitometry analysis of Piezo1 protein abundance in the kidney of sham, 3UUO, and 7UUO mice. Data are shown as mean ± SEM (*n* = 4 in each group). (**E**) mRNA levels of Piezo1, TRPC1, TRPC6, and TRPV4 in the kidney of sham, 3UUO, and 7UUO mice. Data are shown as mean ± SEM (*n* = 4 in each group). (**F**) Colocalization of Piezo1 (red) and AQP1 (green) or AQP2 (green) in the proximal tubules or inner medullary collecting ducts of sham and 7UUO mice, detected by immunofluorescence. Scale bars: 500 μm, 208 μm (insets). *P < 0.05 when compared with sham mice by 1-way ANOVA with Student-Newman-Keuls test. UUO, unilateral ureteral obstruction; 3UUO, UUO for 3 days; 7UUO, UUO for 7 days; TRPC, transient receptor potential canonical; TRPV, transient receptor potential vanilloid.

UUO not only causes elevation of tubular intraluminal pressure; persistent and unrelieved obstruction also leads to kidney interstitial fibrosis. The protein expression of Piezo1 in the kidney of mice started increasing at 30 minutes and significantly increased at the third hour after UUO (Figure 2, A and B), likely due to increased intraluminal pressure. At the third and seventh day after UUO, the protein abundance of Piezo1 was markedly increased to 4- and 7.3-fold, respectively, in the obstructed kidneys, compared with sham mice (Figure 2, C and D). Consistent with this, mRNA levels of Piezo1 were also increased approximately 4.5- and 10-fold at the third and seventh day compared with sham levels (Figure 2E). In contrast, mRNA levels of transient receptor potential canonical 1 (TRPC1) and transient receptor potential vanilloid 4 (TRPV4), capable of responding to membrane stretching, were decreased or unchanged in the obstructed kidneys (Figure 2E). TRPC6 mRNA expression levels were increased 1.7- and 4-fold at the third and seventh day after UUO, respectively (Figure 2E). Immunofluorescence showed little labeling of Piezo1 in tubular epithelia in sham mouse kidneys (Figure 2F), consistent with a previous study (31); however, the labeling intensity of Piezo1 was markedly stronger in tubular segments of the kidney in mice with 7UUO (Figure 2F) than that in sham, especially in the proximal tubular epithelial cells where AQP1 was located. These data indicated that renal fibrosis was associated with upregulated expression of Piezo1.

### Inhibition of Piezo1 prevents UUO-induced kidney fibrosis.

**Figure 3:**
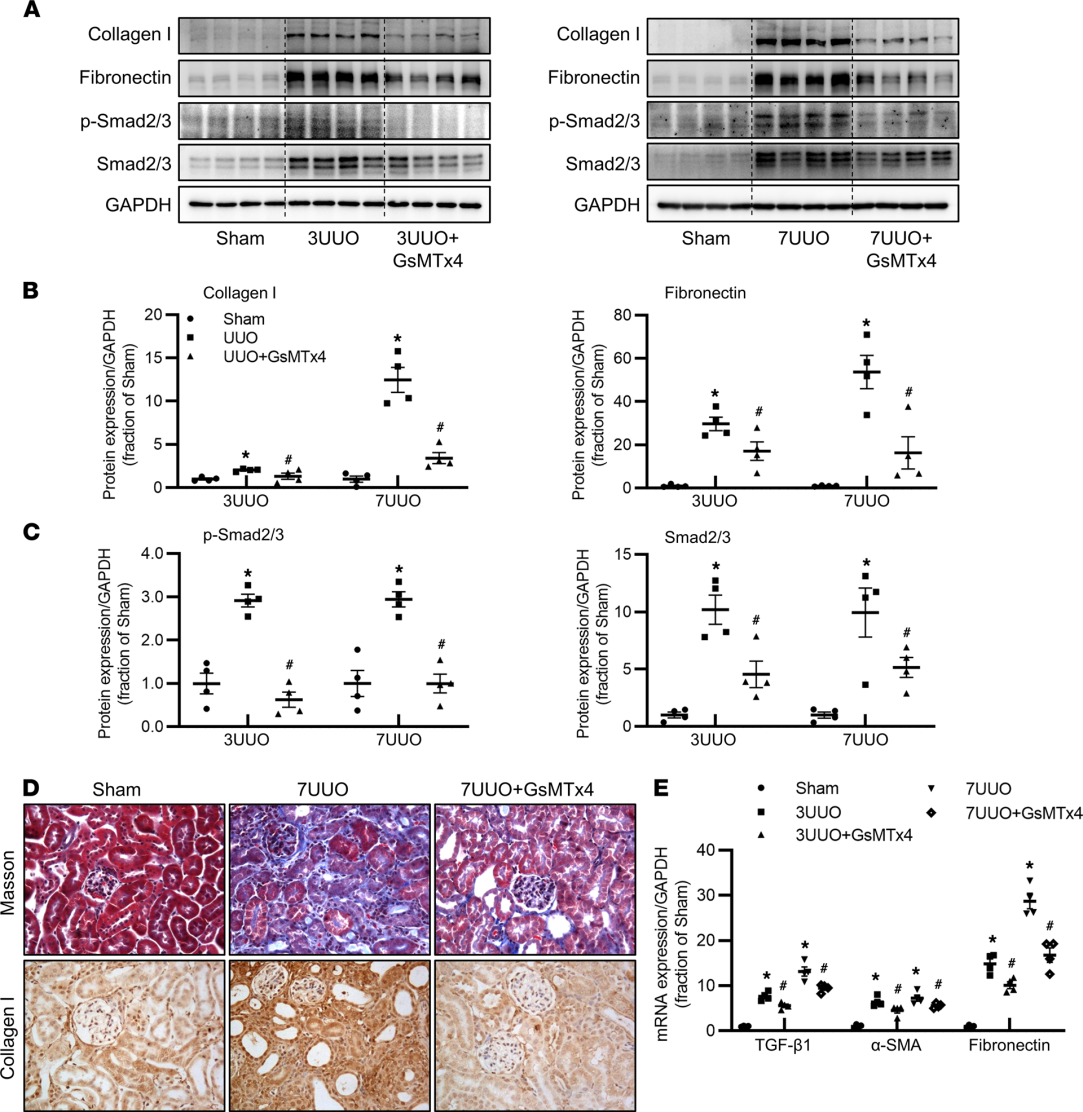
Inhibition of Piezo1 prevents UUO-induced kidney fibrosis. (**A**–**C**) Representative immunoblots and corresponding densitometry analysis of collagen I, fibronectin, p-Smad2/3, and Smad2/3 protein abundance in the kidney of sham, 3UUO, 3UUO+GsMTx4, 7UUO, and 7UUO+GsMTx4 mice. Data are shown as mean ± SEM (*n* = 4 in each group). (**D**) Photomicrographs of Masson’s staining and immunohistochemistry of collagen I in the kidney of sham, 7UUO, and 7UUO+GsMTx4 mice (original magnification, 400×). (**E**) mRNA levels of TGF-β_1_, α-SMA, and fibronectin in the kidney of sham, 3UUO, 3UUO+GsMTx4, 7UUO, and 7UUO+GsMTx4 mice. Data are shown as mean ± SEM (*n* = 4 in each group); *P < 0.05 when compared with sham mice and ^#^*P* < 0.05 when compared with 3UUO or 7UUO mice by 1-way ANOVA with Student-Newman-Keuls test. p-, phosphorylated; TGF-β1, transforming growth factor-β_1_; SMA, smooth muscle actin.

Next, we investigated whether inhibition of Piezo1 improved UUO-induced renal fibrosis. Semiquantitative immunoblotting demonstrated that the protein abundance of fibrotic markers fibronectin and collagen I was dramatically increased in the kidney of mice after 3 days and 7 days UUO, which was markedly reversed by Piezo1-nonspecific inhibitor GsMTx4 treatment (Figure 3, A and B). Piezo1 protein expression was markedly increased in the kidneys of 3UUO and 7UUO mice, and GsMTx4 could partially decrease Piezo1 expression in 3UUO but not in 7UUO mice (Supplemental Figure 1, A–D; supplemental material available online with this article; https://doi.org/10.1172/jci.insight.152330DS1). In canonical TGF-β_1_ signaling, Smad2 and Smad3 are 2 key downstream mediators that are highly activated in the fibrotic kidney (32). In line with this, inhibition of Piezo1 by GsMTx4 also markedly decreased the protein expression of phosphorylated Smad2 at Ser465/467, phosphorylated Smad3 at Ser423/425 and total Smad2/3 in the kidney of mice with 3UUO and 7UUO (Figure 3, A and C). Amelioration of fibrosis by GsMTx4 was confirmed by Masson’s trichrome staining and immunohistochemistry for collagen I. Compared with sham mice, 7UUO caused more fibrotic lesions in tubulointerstitial areas and more extensive and stronger staining of collagen I, which was greatly prevented by GsMTx4 (Figure 3D). Consistent with this, increased mRNA expression levels of TGF-β_1_, α–smooth muscle actin (α-SMA), and fibronectin in the kidney of mice with 3UUO and 7UUO were partially suppressed by inhibition of Piezo1 (Figure 3E). These results suggested that inhibition of Piezo1 by GsMTx4 at least partially prevented UUO-induced tubulointerstitial fibrosis in the obstructed kidneys.

### Inhibition of Piezo1 prevents folic acid–induced kidney fibrosis.

**Figure 4:**
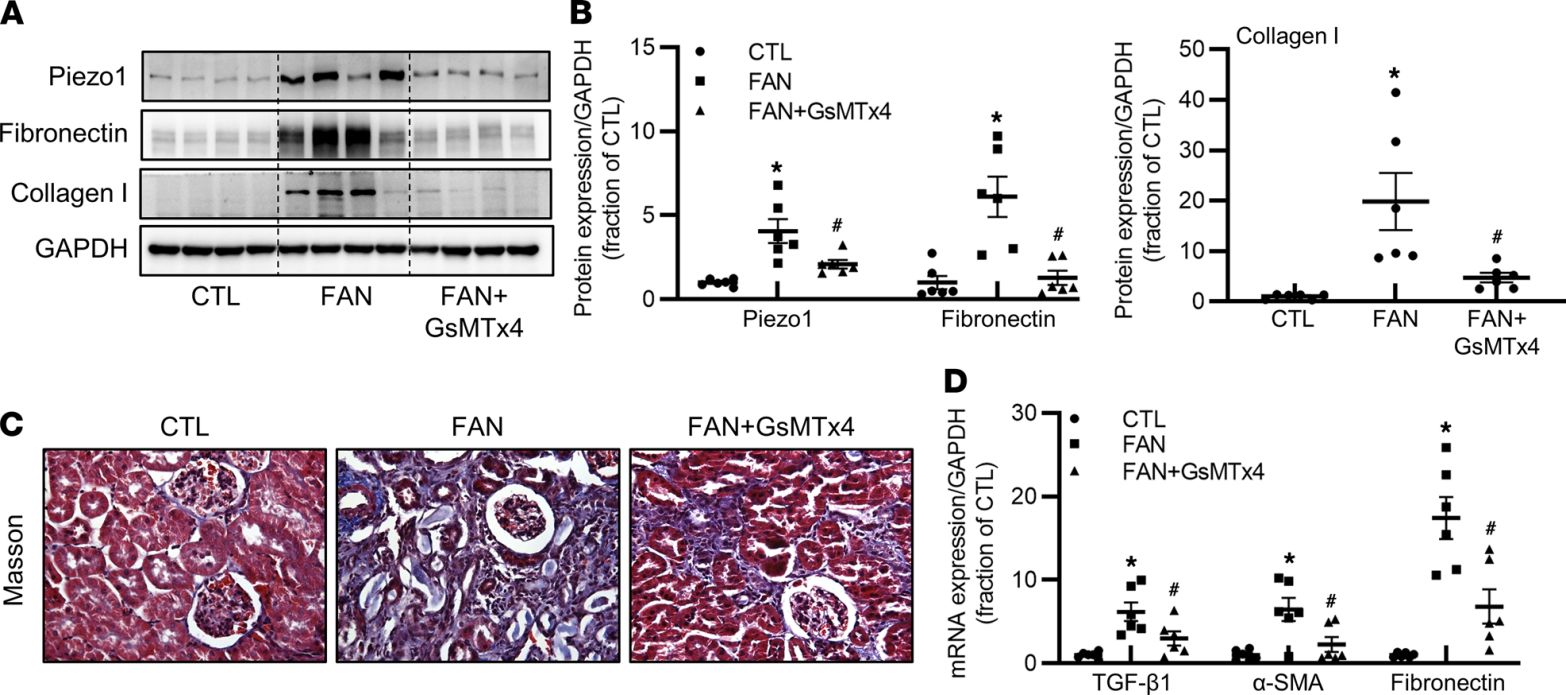
Inhibition of Piezo1 prevents folic acid–induced kidney fibrosis. (**A** and **B**) Representative immunoblots and corresponding densitometry analysis of Piezo1, fibronectin, and collagen I protein abundance in the kidney of CTL, FAN, and FAN+GsMTx4 mice. Data are shown as mean ± SEM (*n* = 5 in CTL group, *n* = 6 in FAN and FAN+GsMTx4 group). (**C**) Photomicrographs of Masson’s staining in the kidney of CTL, FAN, and FAN+GsMTx4 mice (original magnification, 400×). (**D**) mRNA levels of TGF-β_1_, α-SMA, and fibronectin in the kidney of CTL, FAN, and FAN+GsMTx4 mice. Data are shown as mean ± SEM (*n* = 5 in CTL group, *n* = 6 in FAN and FAN+GsMTx4 group). *P < 0.05 when compared with CTL mice and ^#^*P* < 0.05 when compared with FAN mice by 1-way ANOVA with Student-Newman-Keuls test. CTL, control; FAN, folic acid nephropathy.

To further investigate whether inhibition of Piezo1 also attenuates fibrosis in a nonobstructive kidney disease, a folic acid–induced nephropathy (FAN) mouse model was set up to exclude the possibility that upregulation of Piezo1 expression is only due to increased intraluminal pressure after UUO. Compared with controls, the protein abundance of Piezo1 in the kidney of mice with FAN was greatly increased, which was clearly prevented by GsMTx4 (Figure 4, A and B). Western blotting demonstrated that the protein abundance of fibronectin and collagen I was increased in the kidney of mice with FAN, which was again reversed by GsMTx4 (Figure 4, A and B). Consistent with this, Masson’s trichrome staining showed more fibrotic lesions in renal tubulointerstitial areas of mice with FAN that were also at least partially prevented by GsMTx4 (Figure 4C). mRNA levels of TGF-β_1_, α-SMA, and fibronectin were significantly increased in the kidney of mice with FAN; GsMTx4 reduced their expression levels (Figure 4D). Taken together, these findings indicated that inhibition of Piezo1 at least partially attenuated tubulointerstitial fibrosis in the kidney.

### Activation of Piezo1 by Yoda1 was associated with increased expression of profibrotic factors.

As GsMTx4 is not a specific inhibitor of Piezo1, the role of Piezo1 in renal fibrosis was further verified by using a Piezo1-specific agonist, Yoda1. Compared with controls, the protein abundance of fibronectin and TGF-β_1_ in the kidney was significantly increased in mice treated with Yoda1, although Piezo1 abundance was unchanged (Supplemental Figure 2, A and B). These data supported a role of Piezo1 activation in renal fibrosis.

### Piezo1 mediates mechanical stretch– or compression-induced profibrotic responses in HK2 cells.

**Figure 5:**
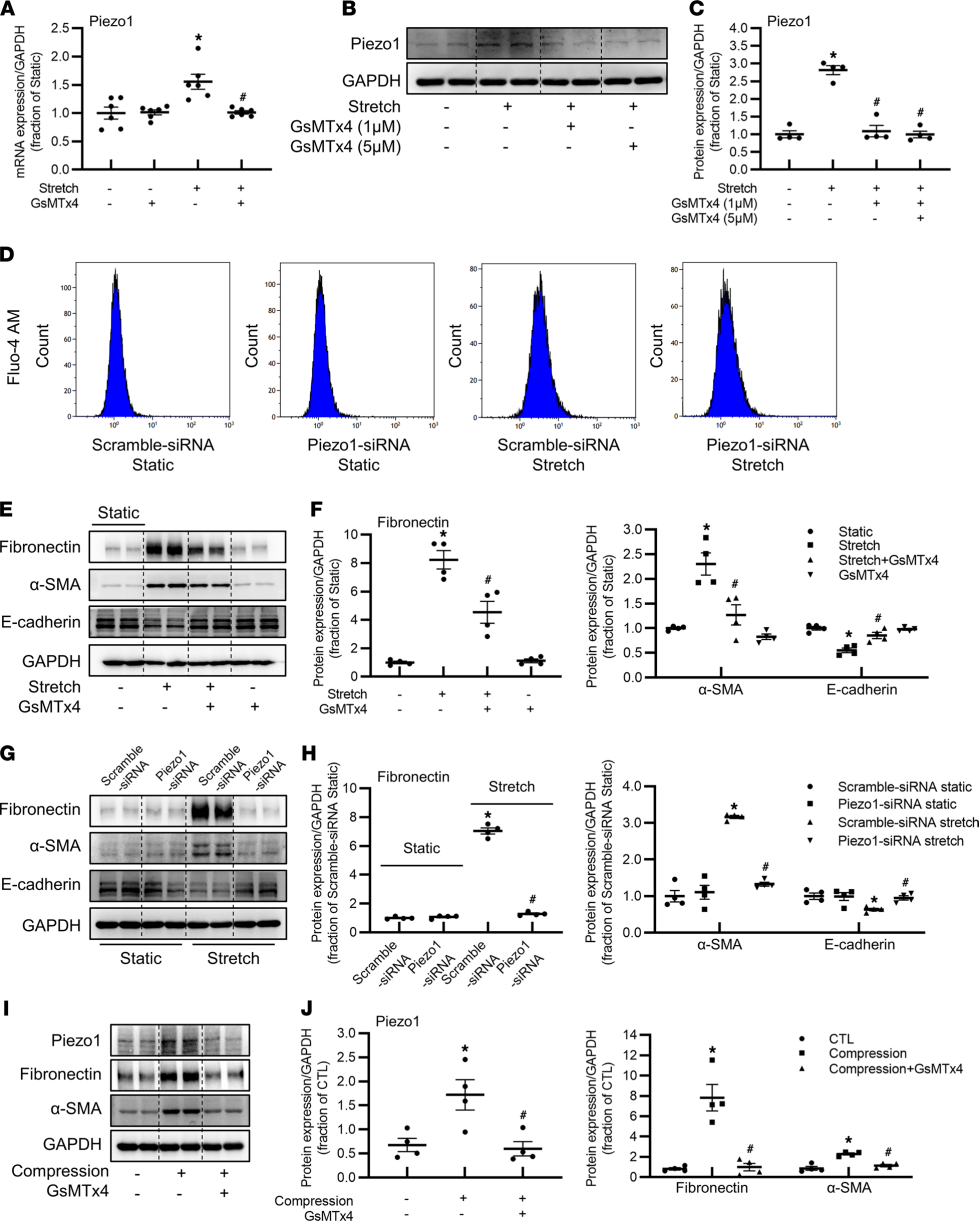
Piezo1 mediates mechanical stretch– or compression-induced profibrotic responses in HK2 cells. (**A**) mRNA level of *Piezo1* in HK2 cells pretreated with 5 μM GsMTx4 followed by cyclic stretch. Data are shown as mean ± SEM (*n* = 6 in each group). (**B** and **C**) Representative immunoblots and corresponding densitometry analysis of Piezo1 protein abundance in HK2 cells pretreated with 1 μM or 5 μM GsMTx4 followed by cyclic stretch. Data are shown as mean ± SEM (*n* = 4 in each group). (**D**) Fluo-4-AM was added to HK2 cells transfected with Piezo1 siRNA followed by cyclic stretch, and the fluorescence signals of calcium were detected by flow cytometry analysis. (**E** and **F**) Representative immunoblots and corresponding densitometry analysis of fibronectin, α-SMA, and E-cadherin protein abundance in HK2 cells pretreated with 5 μM GsMTx4 followed by cyclic stretch. Data are shown as mean ± SEM (*n* = 4 in each group). *P < 0.05 when compared with static and ^#^*P* < 0.05 when compared with stretch by 1-way ANOVA with Student-Newman-Keuls test. (**G** and **H**) Representative immunoblots and corresponding densitometry analysis of fibronectin, α-SMA, and E-cadherin protein abundance in HK2 cells transfected with Piezo1 siRNA followed by cyclic stretch. Data are shown as mean ± SEM (*n* = 4 in each group). *P < 0.05 when compared with scramble siRNA static and ^#^*P* < 0.05 when compared with scramble siRNA stretch by 1-way ANOVA with Student-Newman-Keuls test. (**I** and **J**) Representative immunoblots and corresponding densitometry analysis of Piezo1, fibronectin, and α-SMA protein abundance in HK2 cells pretreated with 5 μM GsMTx4 followed by 15 mmHg compression for 24 hours. Data are shown as mean ± SEM (*n* = 4 in each group). *P < 0.05 when compared with CTL and ^#^*P* < 0.05 when compared with compression by 1-way ANOVA with Student-Newman-Keuls test.

UUO elevates membrane tension of tubular epithelium due to the accumulation of tubular fluid and the deposition of ECM in interstitial spaces, if continued for a long time. Increased tubular stretch is an inducer of profibrotic phenotypic changes in tubular epithelial cells (6). Whether Piezo1, as a mechanosensitive channel, mediated stretch-induced profibrotic effects was investigated in human proximal tubule HK2 cells. First, by quantitative reverse transcription PCR, we found mRNA expression level of Piezo1 in HK2 cells was high (Supplemental Figure 3A). A cyclic stretch sustained for 24 hours at 20% elongation increased *Piezo1* mRNA levels about 1.5-fold in HK2 cells, which were suppressed by GsMTx4 at 5 μM (Figure 5A). Consistent with this, a cyclic stretch induced a 3-fold increase in abundance of Piezo1 protein expression, which was markedly suppressed by GsMTx4 at 1 and 5 μM (Figure 5, B and C). Inhibition of Piezo1 by siRNA (Figure 5D) or GsMTx4 (Supplemental Figure 3B) completely suppressed the elevated intracellular calcium ([Ca^2+^]_i_) levels invoked by mechanical stretch, indicating that Piezo1 channels were able to be activated by mechanical stretch in HK2 cells. Interestingly, mechanical stretch caused increased protein abundance of fibronectin and α-SMA, accompanied with decreased protein expression of an epithelial marker, E-cadherin, in HK2 cells, both of which were partially prevented by Piezo1 inhibitor GsMTx4 (Figure 5, E and F), while GsMTx4 treatment did not cause any changes in expression of these proteins in controls (Figure 5, E and F). To further examine the role of Piezo1 in stretch-induced profibrotic alterations in HK2 cells, Piezo1 siRNA was used (Supplemental Figure 3, C and D). Piezo1 knockdown by siRNA completely prevented cyclic stretch–induced increased protein abundance of fibronectin and α-SMA and reversed protein expression of E-cadherin to control levels (Figure 5, G and H). In the UUO or FAN model, tubular epithelia were also subjected to compression from increased intraluminal pressure or accumulated ECM. HK2 cells were subjected to mechanical compression (15 mmHg, at a frequency of 1 Hz) using Flexcell-5000C Compression Plus System. Mechanical compression was associated with increased expression of Piezo1, fibronectin, and α-SMA in HK2 cells, while GsMTx4 could partially prevent these profibrotic alterations (Figure 5, I and J). These findings suggested that Piezo1 was involved in mechanical stretch– or compression-induced profibrotic alterations in HK2 cells.

### Matrix stiffness is associated with activation of Piezo1 in proximal tubular cells.

**Figure 6:**
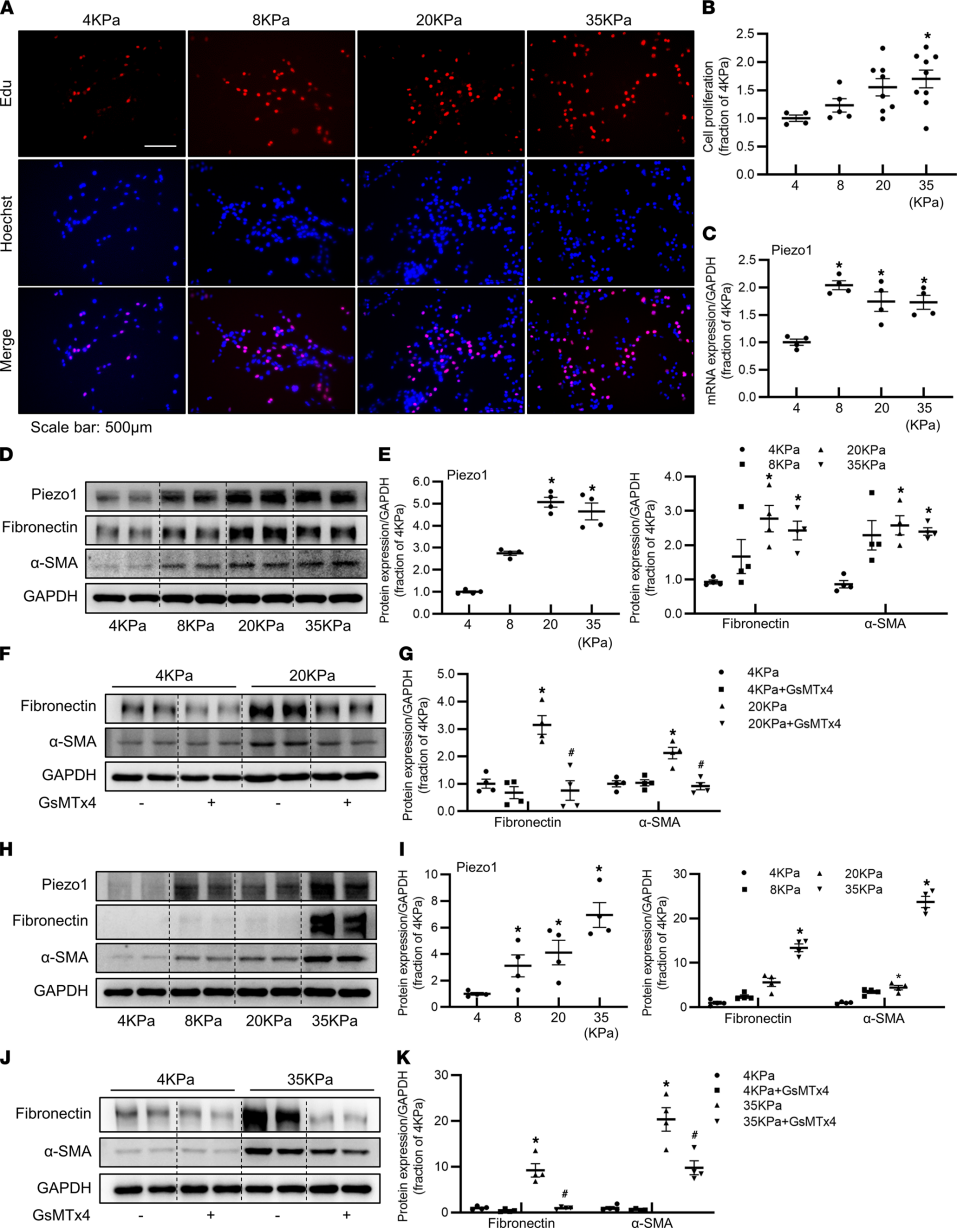
Piezo1 senses matrix stiffness and is involved in increased matrix stiffness–induced profibrotic responses in HK2 cells and mouse proximal tubular cells. (**A** and **B**) Cell proliferation of HK2 cells cultured on polyacrylamide hydrogels with different stiffness, assessed by EdU. Scale bar: 500 μm. Data are shown as mean ± SEM (*n* = 4 in 4 kPa group, *n* = 5 in 8 kPa group, *n* = 8 in 20 kPa group, *n* = 9 in 35 kPa group). (**C**) *Piezo1* mRNA level of HK2 cells cultured on polyacrylamide hydrogels with different stiffness. Data are shown as mean ± SEM (*n* = 4 in each group). (**D** and **E**) Representative immunoblots and corresponding densitometry analysis of Piezo1, fibronectin, and α-SMA protein abundance in HK2 cells cultured on polyacrylamide hydrogels with different stiffness. Data are shown as mean ± SEM (*n* = 4 in each group). (**F** and **G**) Representative immunoblots and corresponding densitometry analysis of fibronectin and α-SMA protein abundance in HK2 cells cultured on 4 kPa and 20 kPa polyacrylamide hydrogels, followed by treatment with GsMTx4. Data are shown as mean ± SEM (*n* = 4 in each group). *P < 0.05 when compared with 4 kPa and ^#^*P* < 0.05 when compared with 20 kPa by 1-way ANOVA with Student-Newman-Keuls test. (**H** and **I**) Representative immunoblots and corresponding densitometry analysis of Piezo1, fibronectin, and α-SMA protein abundance in primary mPTCs cultured on polyacrylamide hydrogels with different stiffness. Data are shown as mean ± SEM (*n* = 4 in each group). (**J** and **K**) Representative immunoblots and corresponding densitometry analysis of fibronectin and α-SMA protein abundance in primary mPTCs cultured on polyacrylamide hydrogels with 4 kPa and 35 kPa stiffness, followed by treatment with GsMTx4. Data are shown as mean ± SEM (*n* = 4 in each group). *P < 0.05 when compared with 4 kPa and ^#^*P* < 0.05 when compared with 35 kPa by 1-way ANOVA with Student-Newman-Keuls test. mPTCs, mouse proximal tubular cells; EdU, 5-ethynyl-2′-deoxyuridine.

Renal fibrosis is characterized by increased tissue stiffness due to excessive ECM deposition in interstitial areas, leading to increased tubular membrane tension. Next, we aimed to investigate whether increased tissue stiffness induced by ECM deposition activated Piezo1 and the potential interaction between Piezo1 and ECM production. Polyacrylamide (PA) hydrogels can simulate the in vivo environment and could be a promising scaffold material, mimicking the natural ECM (33, 34). HK2 cells were cultured on PA hydrogels with the modulus of 4 kPa to 35 kPa, which encompasses the stiffness range from normal to fibrotic stiffness of kidney cortex. An EdU assay showed that proliferation of HK2 cells was greatly increased with stiffness of PA hydrogels (Figure 6, A and B). With increases in stiffness, both protein and mRNA expressions of Piezo1 were significantly increased in HK2 cells cultured on PA hydrogels. Stiffness modulus of 8, 20, or 35 kPa caused about 2-fold increases of *Piezo1* mRNA expression (Figure 6C). The protein abundance of Piezo1 was increased about 3-fold under 8 kPa, and approximate 5-fold increases were observed under 20 and 35 kPa in HK2 cells (Figure 6, D and E). With increases in stiffness, the protein abundance of fibronectin and α-SMA was significantly increased in HK2 cells (Figure 6, D and E). At 20 kPa modulus, increased fibronectin and α-SMA was inhibited by GsMTx4 (Figure 6, F and G). The upregulation of Piezo1, fibronectin, and α-SMA induced by stiffness was also observed in primary cultured mPTCs (Figure 6, H and I). Again, GsMTx4 prevented such a profibrotic response (Figure 6, J and K). These data suggested that Piezo1 was capable of sensing matrix stiffness, and increased matrix stiffness–induced profibrotic alterations in tubular cells were, at least partially, depending of Piezo1 activation.

### Piezo1 mediated TGF-β_1_–induced profibrotic responses in proximal tubular cells.

**Figure 7:**
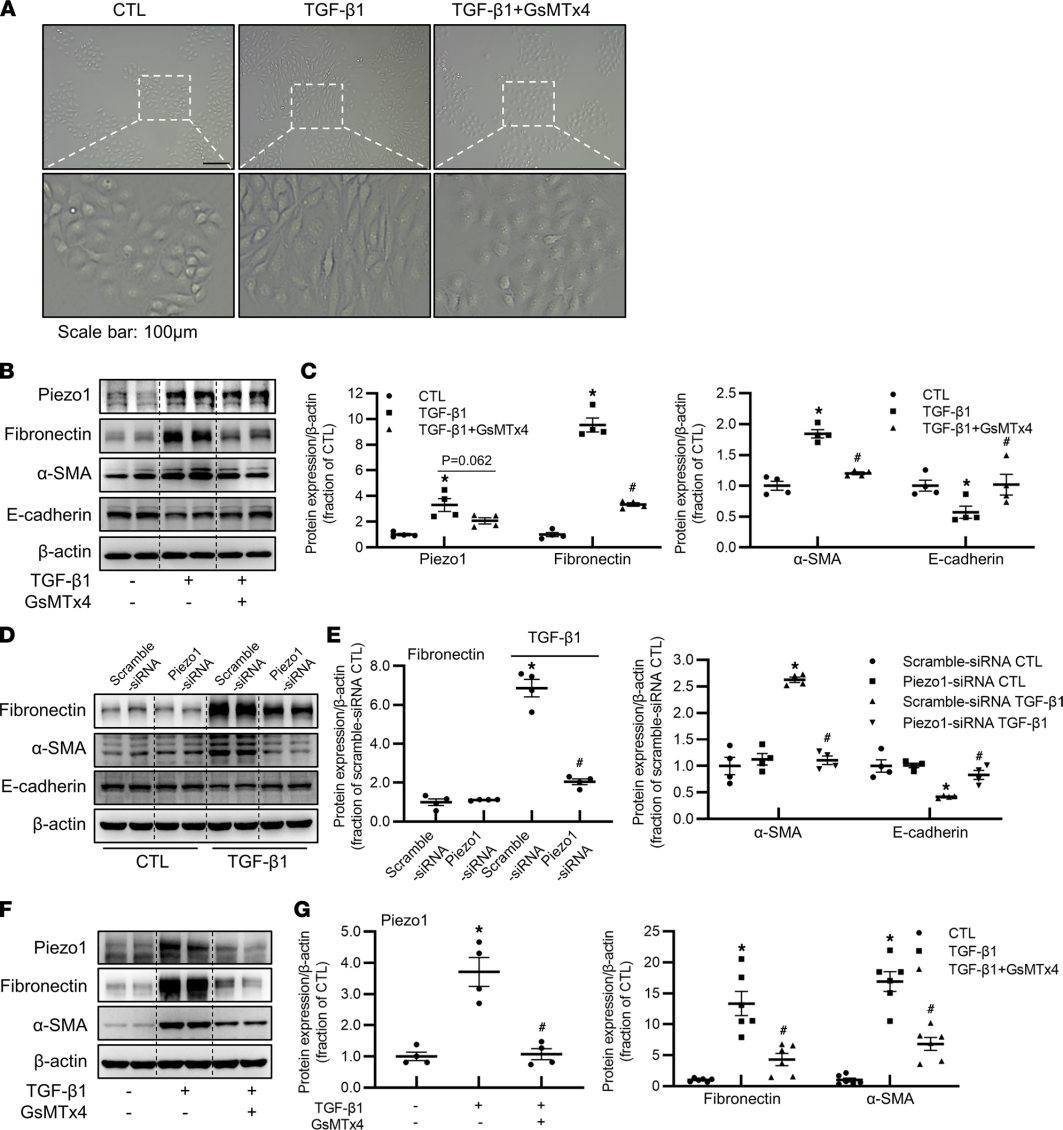
Piezo1 is involved in TGF-β_1_–induced profibrotic responses in HK2 cells and mPTCs. (**A**) Morphologic changes of HK2 cells pretreated with GsMTx4 followed by TGF-β_1_ treatment for 48 hours. Scale bar: 100 μm. (**B** and **C**) Representative immunoblots and corresponding densitometry analysis of Piezo1, fibronectin, α-SMA, and E-cadherin protein abundance in HK2 cells pretreated with GsMTx4 followed by TGF-β_1_ treatment for 48 hours. Data are shown as mean ± SEM (*n* = 4 in each group). *P < 0.05 when compared with CTL and ^#^*P* < 0.05 when compared with TGF-β_1_ by 1-way ANOVA with Student-Newman-Keuls test. (**D** and **E**) Representative immunoblots and corresponding densitometry analysis of fibronectin, α-SMA, and E-cadherin protein abundance in HK2 cells transfected with Piezo1 siRNA followed by TGF-β_1_ treatment for 48 hours. Data are shown as mean ± SEM (*n* = 4 in each group). *P < 0.05 when compared with scramble siRNA CTL and ^#^*P* < 0.05 when compared with scramble siRNA TGF-β_1_ by 1-way ANOVA with Student-Newman-Keuls test. (**F** and **G**) Representative immunoblots and corresponding densitometry analysis of Piezo1, fibronectin, and α-SMA protein abundance in primary cultured mPTCs, pretreated with GsMTx4 followed by TGF-β_1_ treatment for 48 hours. Data are shown as mean ± SEM (*n* = 6 in each group). *P < 0.05 when compared with CTL and ^#^*P* < 0.05 when compared with TGF-β_1_ by 1-way ANOVA with Student-Newman-Keuls test.

TGF-β_1_ induces tubular EMT and excessive production and deposition of ECM in tubulointerstitium (6), contributing to the early development and progression of renal interstitial fibrosis. We next examined the role of Piezo1 in TGF-β_1_–induced tubular profibrotic alterations in HK2 cells. TGF-β_1_ treatment caused morphologic changes in HK2 cells, which were clearly prevented by GsMTx4 (Figure 7A). Consistent with this, Western blots revealed that GsMTx4 prevented upregulation in protein expression of fibronectin and α-SMA and reversed decreased protein abundance of E-cadherin in HK2 cells treated with TGF-β_1_ (Figure 7, B and C). TGF-β_1_ induced about 3-fold increase in Piezo1 protein expression, which was partially prevented by GsMTx4 (*P* = 0.06) (Figure 7, B and C). Piezo1 silencing with siRNA markedly prevented increases in protein expression of fibronectin and α-SMA and a decrease in E-cadherin in HK2 cells treated with TGF-β_1_ (Figure 7, D and E). In mPTCs, TGF-β_1_ clearly increased protein expression of Piezo1, fibronectin, and α-SMA, which was inhibited by GsMTx4 (Figure 7, F and G). Taken together, these data suggested an involvement of Piezo1 in TGF-β_1_–induced profibrotic effects.

Recent data suggest a close association between TGF-β_1_ and stiff environment in development of fibrosis (35). Interestingly, with activation of Piezo1, HK2 cells seemed sensitive to TGF-β_1_ treatment when cultured on stiffer PA hydrogels. After TGF-β_1_ treatment, the protein abundance of fibronectin was increased more in HK2 cells cultured on 20 kPa PA hydrogels than those on 4 kPa PA hydrogels (Supplemental Figure 4, A and B). This suggests a positive synergy between matrix stiffening and TGF-β_1_ in inducing Piezo1 activation in fibrosis.

### Piezo1 activation by Yoda1 induces profibrotic responses in proximal tubular cells.

**Figure 8:**
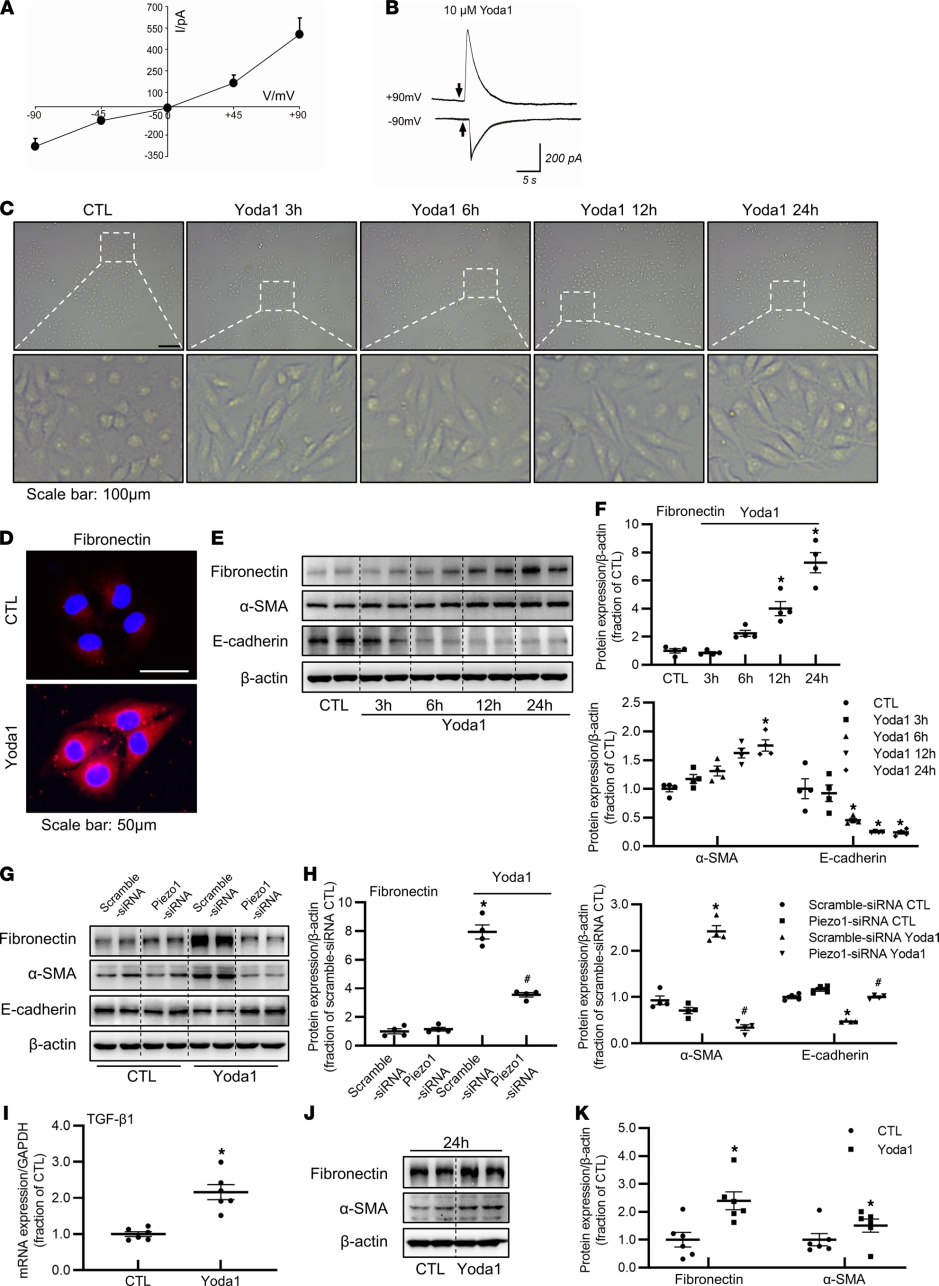
Activation of Piezo1 by an agonist, Yoda1, induces profibrotic responses in HK2 cells and mPTCs. (**A** and **B**) Yoda1 induced the cationic currents in HK2 cells. (**A**) I-V curve exhibited the currents activated by 10 μM Yoda1 recorded at different membrane potentials in voltage-clamp mode. (**B**) Representative traces: recorded at –90 and +90 mV by puffing 10 μM Yoda1 (short black arrow) for 350 ms to HK2 cells. (**C**) Morphologic changes of HK2 cells treated with Yoda1 for 3 hours, 6 hours, 12 hours, and 24 hours. Scale bar: 100 μm. (**D**) Immunofluorescence of fibronectin in HK2 cells treated with Yoda1 for 24 hours. Scale bar: 50 μm. (**E** and **F**) Representative immunoblots and corresponding densitometry analysis of fibronectin, α-SMA, and E-cadherin protein abundance in HK2 cells treated with Yoda1 for 3 hours, 6 hours, 12 hours, and 24 hours. Data are shown as mean ± SEM (*n* = 4 in each group). *P < 0.05 when compared with CTL by 1-way ANOVA with Student-Newman-Keuls test. (**G** and **H**) Representative immunoblots and corresponding densitometry analysis of fibronectin, α-SMA, and E-cadherin protein abundance in HK2 cells transfected with Piezo1 siRNA followed by Yoda1 treatment for 24 hours. Data are shown as mean ± SEM (*n* = 4 in each group). *P < 0.05 when compared with scramble siRNA CTL and ^#^*P* < 0.05 when compared with scramble siRNA Yoda1 by 1-way ANOVA with Student-Newman-Keuls test. (**I**) mRNA expression of TGF-β_1_ in HK2 cells treated with Yoda1 for 24 hours. Data are shown as mean ± SEM (*n* = 6 in each group). *P < 0.05 when compared with CTL by unpaired Student’s *t* test. (**J** and **K**) Representative immunoblots and corresponding densitometry analysis of fibronectin and α-SMA protein abundance in primary cultured mPTCs treated with Yoda1 for 24 hours. Data are shown as mean ± SEM (*n* = 6 in each group). *P < 0.05 when compared with CTL by unpaired Student’s *t* test. CTL, control; SMA, smooth muscle actin; TGF-β1, transforming growth factor-β_1_; mPTCs: mouse proximal tubular cells.

Mechanical stretch, compression, increased matrix stiffness, or TGF-β_1_, by activating Piezo1, leads to profibrotic alterations in HK2 cells and mPTCs, so we then asked whether direct stimulation by a Piezo1-specific agonist, Yoda1, causes profibrotic responses. First, the activity of Piezo1 was examined by whole-cell recording and cationic current was recorded. Yoda1 could induce marked cationic currents under different potentials as shown in the current-voltage relationship (I-V) (Figure 8A). Under a high holding potential, puffing 10 μM Yoda1 caused a marked current in HK2 cells (Figure 8B). These data confirmed expression of Piezo1 in HK2 cells, and Piezo1 functioned well under Yoda1 stimulation.

In a time-dependent manner, Yoda1 gradually caused morphologic changes in HK2 cells. HK2 cells in controls showed the typical cobblestone morphology of epithelial cells, while HK2 cells treated with Yoda1 exhibited elongation and hypertrophy after 24-hour treatment (Figure 8C). After 24-hour treatment with Yoda1, immunofluorescence showed a marked increase in fibronectin labeling intensity (Figure 8D). Consistent with morphological alterations, Western blot demonstrated a time-dependent increase in the protein abundance of fibronectin and α-SMA, as well as a decrease in the protein expression of E-cadherin in HK2 cells treated with Yoda1 (Figure 8, E and F), whereas Piezo1 silence with siRNA markedly prevented Yoda1-induced profibrotic response (Figure 8, G and H). Interestingly, Yoda1 stimulation caused a 2-fold increase in mRNA expression levels of TGF-β_1_ in HK2 cells (Figure 8I) and a 1.7-fold increase in protein abundance of TGF-β_1_ in the kidney of mice (Supplemental Figure 2, A and B), indicating that Piezo1 activation may induce TGF-β_1_ synthesis and secretion. The profibrotic alterations caused by activation of Piezo1 were also seen in mPTCs treated with Yoda1 (Figure 8, J and K). Taken together, these results suggested that activation of Piezo1 by Yoda1 directly induced profibrotic phenotype changes in tubular cells and that activation of Piezo1 may be an important step in the development of fibrosis.

### Activation of Piezo1 induces calcium influx and enhances interactions among calpain2, integrin β_1_, and fibronectin.

**Figure 9:**
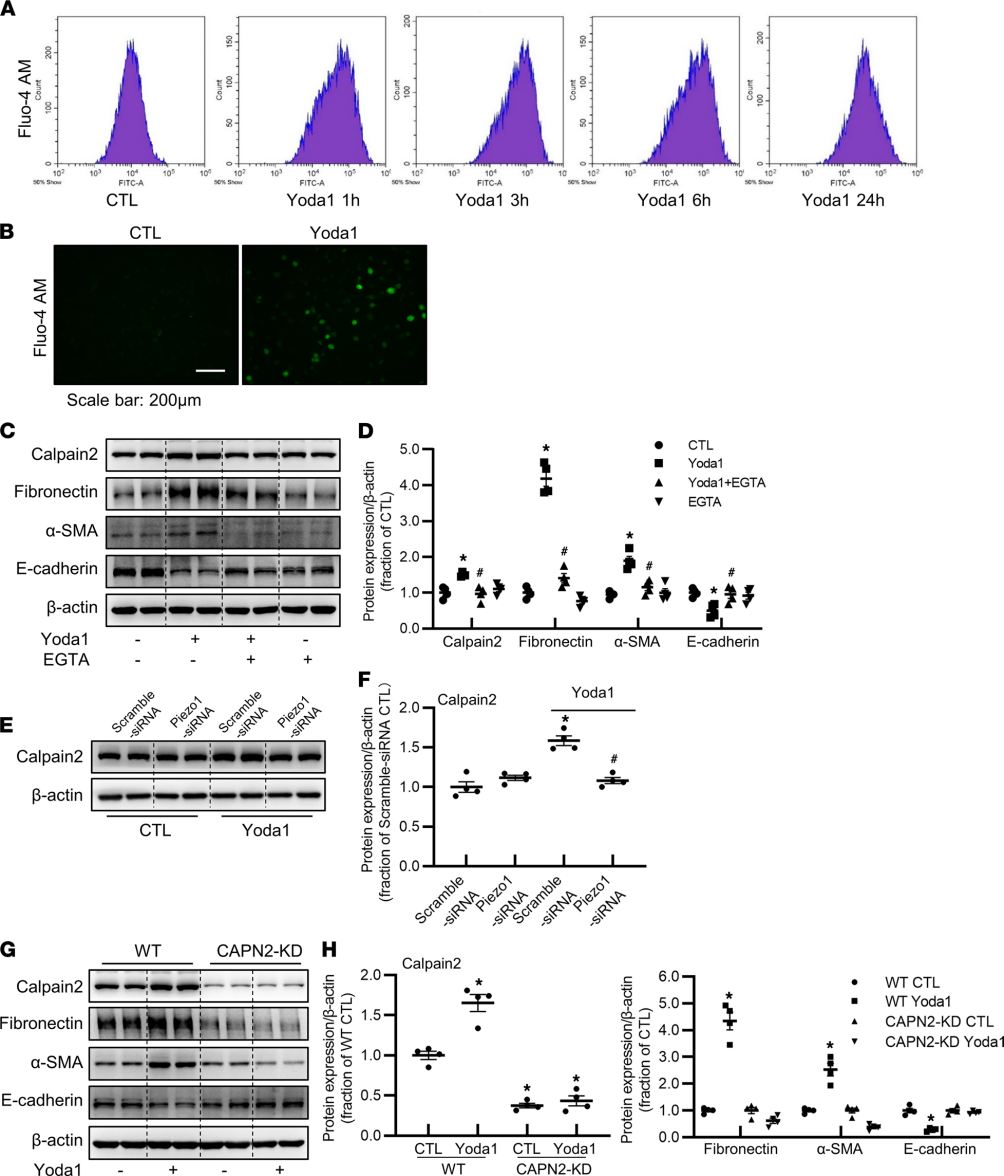
Yoda1-induced calpain2 activation promotes profibrotic responses in HK2 cells. (**A** and **B**) Fluo-4-AM was added to HK2 cells treated with Yoda1 for 1 hour, 3 hours, 6 hours, and 24 hours, and the fluorescence signals of Ca^2+^ were detected by flow cytometry analysis and fluorescence microscope. Scale bar: 200 μm. “50% Show” means that flow cytometry data are presented based on 10,000 cells of 20,000 cells analyzed. (**C** and **D**) Representative immunoblots and corresponding densitometry analysis of calpain2, fibronectin, α-SMA, and E-cadherin protein abundance in HK2 cells pretreated with 2 mM EGTA followed by Yoda1 treatment for 24 hours. Data are shown as mean ± SEM (*n* = 4 in each group). *P < 0.05 when compared with CTL and ^#^*P* < 0.05 when compared with Yoda1 by 1-way ANOVA with Student-Newman-Keuls test. (**E** and **F**) Representative immunoblots and corresponding densitometry analysis of calpain2 protein abundance in HK2 cells transfected with Piezo1 siRNA followed by Yoda1 treatment for 24 hours. Data are shown as mean ± SEM (*n* = 4 in each group). *P < 0.05 when compared with scramble siRNA CTL and ^#^*P* < 0.05 when compared with scramble siRNA Yoda1 by 1-way ANOVA with Student-Newman-Keuls test. (**G** and **H**) Representative immunoblots and corresponding densitometry analysis of calpain2, fibronectin, α-SMA, and E-cadherin protein abundance in WT HK2 cells and CAPN2-KD HK2 cells treated with Yoda1 for 24 hours. Data are shown as mean ± SEM (*n* = 4 in each group). *P < 0.05 when compared with CTL by 1-way ANOVA with Student-Newman-Keuls test. CAPN2-KD, calpain2 knockdown.

Next, we investigated the potential molecular mechanisms by which Piezo1 mediates renal fibrosis. Activation of Piezo1 by Yoda1 induced markedly increased calcium influx in HK2 cells (Figure 9, A and B), consistent with previous studies. Calpain2, a calcium-dependent protease, is a member of the calpain families, which can be activated by increased intracellular calcium. Inhibition of Piezo1 by Piezo siRNA or GsMTx4 can reduce calpain activity in HepG2 cells or PC12 cells (36, 37). The calcium/calpain2 pathway was thus examined in our study. Yoda1 increased protein abundance of calpain2 in HK2 cells (Figure 9, C–F). Piezo1 inhibition by siRNA attenuated Yoda1-induced upregulation of calpain2 (Figure 9, E and F), indicating that calpain2 was an important downstream target of Piezo1 (36–38). Piezo1-mediated calcium response seems largely dependent on extracellular calcium levels. When extracellular calcium was chelated by EGTA, increased calpain2 protein expression and profibrotic changes induced by Yoda1 were significantly attenuated (Figure 9, C and D), indicating that increased calcium influx mediated by Piezo1 may activate calpain2, which was involved in Yoda1-induced profibrotic effects. To further investigate the role of calpain2 in Piezo1 activation–induced profibrotic alterations in HK2 cells, calpain2 was knocked down by using CRISPR/Cas9-guided genome editing (Figure 9, G and H). Compared with WT HK2 cells, the protein abundance of fibronectin and α-SMA was greatly decreased in CAPN2-KD HK2 cells after Yoda1 treatment, while E-cadherin protein expression was markedly upregulated (Figure 9, G and H), indicating a suppression of profibrotic response caused by calpain2 knockdown.

**Figure 10:**
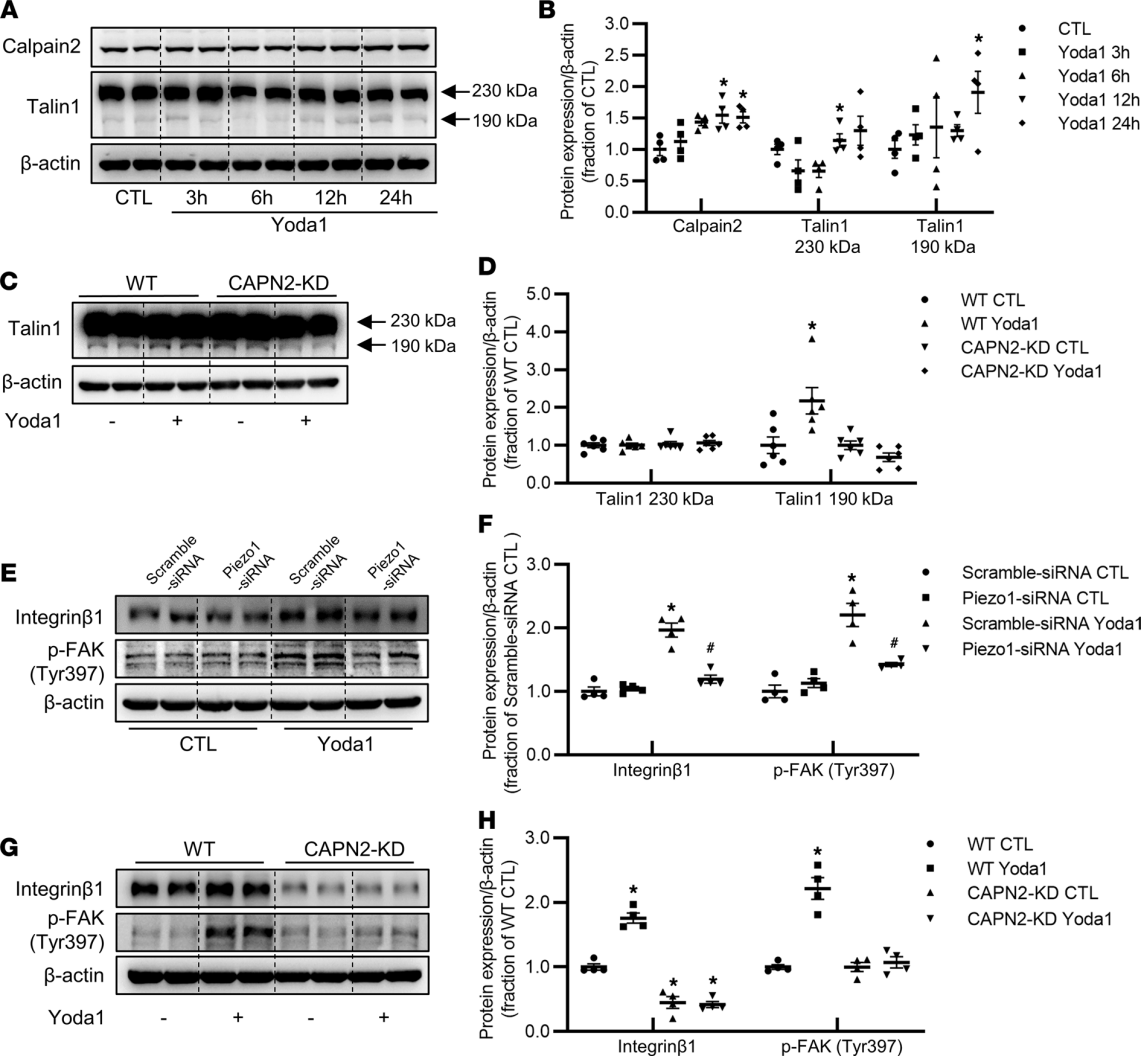
Yoda1 activates the calpain2/talin1/integrin β_1_ pathway in HK2 cells. (**A** and **B**) Representative immunoblots and corresponding densitometry analysis of calpain2 and talin1 protein abundance in HK2 cells treated with Yoda1 for 3 hours, 6 hours, 12 hours, and 24 hours. Data are shown as mean ± SEM (*n* = 4 in each group). *P < 0.05 when compared with CTL by 1-way ANOVA with Student-Newman-Keuls test. (**C** and **D**) Representative immunoblots and corresponding densitometry analysis of talin1 protein abundance in WT HK2 cells and CAPN2-KD HK2 cells treated with Yoda1 for 24 hours. Data are shown as mean ± SEM (*n* = 6 in each group). *P < 0.05 when compared with CTL by 1-way ANOVA with Student-Newman-Keuls test. (**E** and **F**) Representative immunoblots and corresponding densitometry analysis of integrin β_1_ and p-FAK (Tyr397) protein abundance in HK2 cells transfected with Piezo1 siRNA followed by Yoda1 treatment for 24 hours. Data are shown as mean ± SEM (*n* = 4 in each group). *P < 0.05 when compared with scramble siRNA CTL and ^#^*P* < 0.05 when compared with scramble siRNA Yoda1 by 1-way ANOVA with Student-Newman-Keuls test. (**G** and **H**) Representative immunoblots and corresponding densitometry analysis of integrin β_1_ and p-FAK (Tyr397) protein abundance in WT HK2 cells and CAPN2-KD HK2 cells treated with Yoda1 for 24 hours. Data are shown as mean ± SEM (*n* = 4 in each group). *P < 0.05 when compared with CTL by 1-way ANOVA with Student-Newman-Keuls test. FAK, focal adhesion kinase.

Adhesion complex protein talin1 regulates integrin affinity and provides a link between integrins and the cytoskeleton (39), and it can be cleaved into active form by calpain2, mediating inside-out integrin β_1_ activation and affinity (40). Western blot showed that the abundance of cleaved talin1 protein (190 kDa) was markedly increased in response to Yoda1 treatment, in association with a persistent increase of calpain2 protein abundance (Figure 10, A and B). In CAPN2-KD HK2 cells, Yoda1 treatment failed to increase protein expression of cleaved talin1 (Figure 10, C and D).

**Figure 11:**
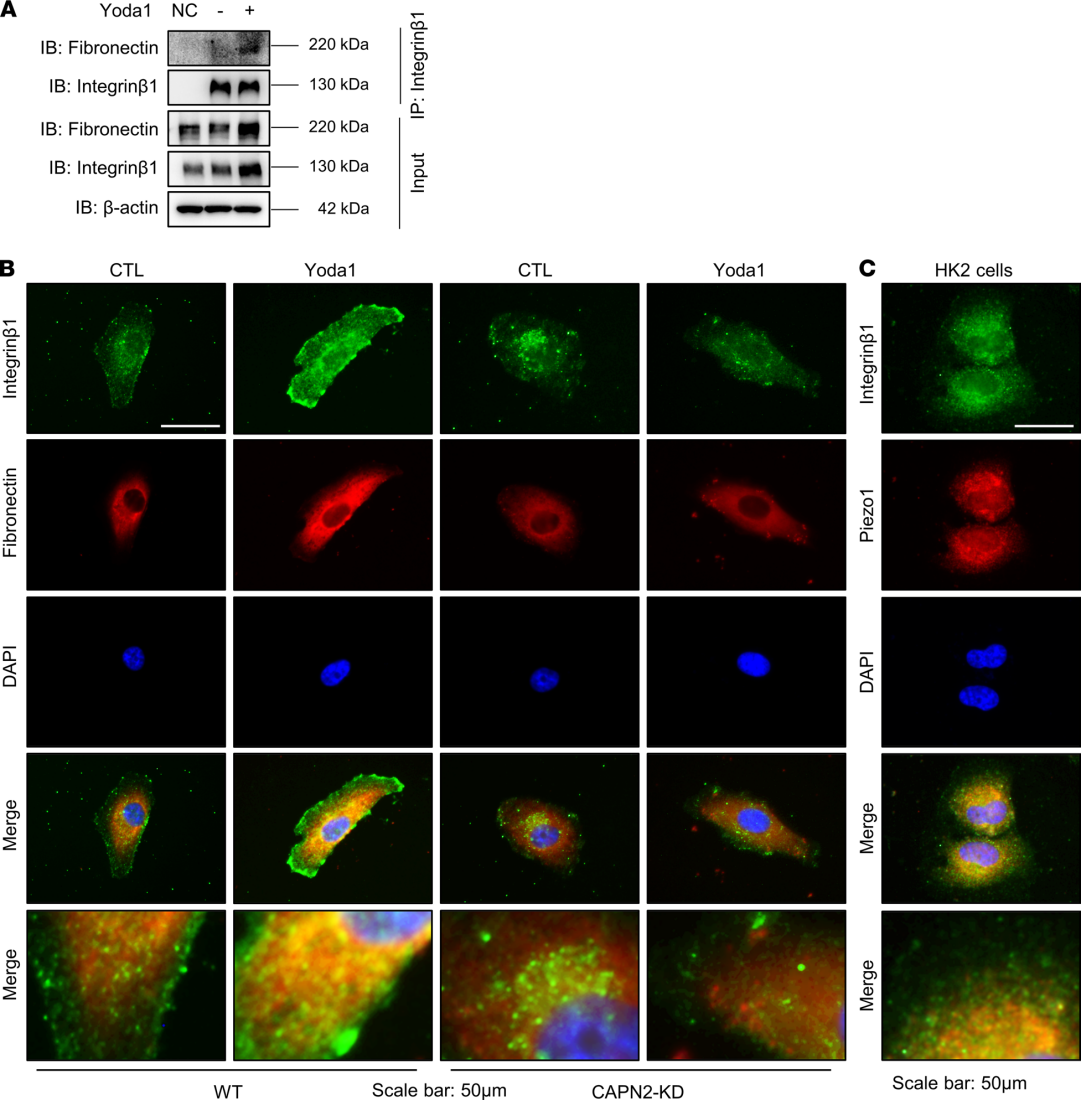
Yoda1 enhances the interaction between fibronectin and integrin β_1_ in HK2 cells. (**A**) Immunoprecipitation assay showing the interaction between fibronectin and integrin β_1_ in WT HK2 cells with or without Yoda1 treatment. (**B**) Immunofluorescence of colocalization of fibronectin and integrin β_1_ in WT HK2 cells and CAPN2-KD HK2 cells treated with or without Yoda1. Scale bars: 50 μm, 12 μm (insets). (**C**) Immunofluorescence of colocalization of Piezo1 and integrin β_1_ in WT HK2 cells. Scale bars: 50 μm, 15 μm (insets).

As cleavage of talin1 by calpain2 causes clustering of integrin on the plasma membrane and increased affinity of integrin β_1_ (41), we next examined potential associations among activated Piezo1, calpain2, and integrin β_1_ expression. Activation of Piezo1 by Yoda1 induced a significant increase in protein abundance of integrin β_1_ in WT HK2 cells, which could be prevented by Piezo1 siRNA (Figure 10, E and F). In CAPN2-KD HK2 cells, the abundance of integrin β_1_ was lower than that in WT HK2 cells and Yoda1 treatment failed to induce integrin β_1_ protein expression (Figure 10, G and H), indicating a potential interaction between calpain2 and integrin β_1_ when Piezo1 was stimulated. In addition, increased matrix stiffness also induced increased expression of calpain2 and integrin β_1_ in HK2 cells and mPTCs, which could be attenuated by GsMTx4 (Supplemental Figure 5, A–D). In mice treated with Yoda1, the protein expression of integrin β_1_ was also increased (Supplemental Figure 2, A and B). These results indicated that Piezo1 activation was probably an upstream event in integrin β_1_ activation and following profibrotic alterations. Co-IP assay showed that Yoda1 treatment induced a close binding of fibronectin to integrin β_1_ (Figure 11A), which was confirmed by immunofluorescence demonstrating marked colocalization of fibronectin and integrin β_1_ in WT HK2 cells treated with Yoda1 (Figure 11B); however, such a colocalization was not found in CAPN2-KD HK2 cells treated with Yoda1 (Figure 11B). Immunofluorescence also demonstrated a prominent colocalization of Piezo1 and integrin β_1_ in HK2 cells (Figure 11C). FAK is a downstream target of integrin signaling, and autophosphorylation of FAK plays an important role in integrin-mediated signal transductions (42). Recent studies showed that the integrin/FAK pathway was involved in the progression of fibrosis (43, 44). In our study, Yoda1 increased protein expression of p-FAK (Tyr397) in HK2 cells, which was prevented by Piezo1 siRNA inhibition (Figure 10, E and F). In CAPN2-KD HK2 cells, Yoda1 could not induce increased expression of p-FAK (Tyr397) (Figure 10, G and H). The protein abundance of integrin β_1_ and p-FAK (Tyr397) was also greatly increased in 7UUO mice, which was markedly reversed by Piezo1 inhibitor GsMTx4 treatment (Supplemental Figure 6, A and B).

Taken together, these results suggested that stimulation of Piezo1 induced activation of calcium/calpain2/talin1 signaling and integrin β_1_/FAK pathway, which may promote profibrotic responses, presumably leading to kidney interstitial fibrosis.

## Discussion

The main finding of the current study is an association between Piezo1 activation and fibrosis in the kidney. In the fibrotic kidneys, tubular Piezo1 protein abundance was markedly increased, while inhibition of Piezo1 by a blocker, GsMTx4, attenuated tubulointerstitial fibrosis induced by UUO or folic acid. We also demonstrated that Piezo1 stimulation by mechanical stretch, compression, stiffness, or an agonist, Yoda1, induced profibrotic alterations in HK2 cells, while TGF-β_1_–induced profibrotic response was partially prevented by inhibition or silence of Piezo1. The involvement of Piezo1 in fibrosis is likely attributed to the activation of calcium/calpain2/integrin β_1_ signaling.

### Kidney fibrosis is associated with increased protein expression of Piezo1.

Piezo1 is essential for transducing externally and internally applied forces at the plasma membrane, by which renal epithelial cells respond to changes in fluid flow, intraluminal pressure, and wall tension (19, 20). In the kidney, Piezo1 mRNA and protein expression are reported in segments of the rodent nephron and collecting duct (31). Piezo1 activity or Piezo1 transcript has been shown in immortalized proximal convoluted tubule cells (19) and the proximal tubule of mouse kidney and HK2 cells (45), respectively. Tubulointerstitial fibrosis is characterized by expansion of the space between tubular basement membrane and peritubular capillaries through ECM deposition (46), due to increased synthesis and inadequate degradation. ECM deposition inevitably leads to increase in peritubular compressional and tensional forces. Excessive ECM accumulation also makes fibrotic regions (cortex, for example) much stiffer (35 kPa) (22) than normal kidney tissue (4 kPa) (23), which may cause an aberrant mechanical, microenvironmental, and stretch/compression stimulus to tubular epithelial cells. All these can be sensed by mechanosensors located on basolateral plasma membrane, among which is Piezo1 (19). In the present study, we found that in both human and mouse fibrotic kidneys, Piezo1 protein expression was much increased when compared with controls, particularly in proximal tubular epithelial cells. It is probably reasonable to believe that Piezo1 may be activated and upregulated when tubular epithelial cells respond to mechanical stress, e.g., fibrosis. Therefore, the observed upregulation of Piezo1 protein expression in the cortex may depend on the physiological or pathophysiological state of the kidney.

Piezo1 activation and upregulation in early time after UUO are likely attributed to tubular mechanical stretch caused by retrograde pressure shifts and urine pooling; while in sustained obstruction, the volume and continued pooling of urine provide major tubular stretch stimuli (47). Therefore, during UUO, tubular epithelial cells, at least partially, through Piezo1, sense stretch or swelling forces in apical plasma membrane and stiff microenvironments in basolateral sides after fibrosis development. Besides, TRP ion channels located in epithelial cells also play important roles in sensing mechanical stimuli. After UUO, mRNA levels of TRPC1 and TRPV4 were slightly decreased or unchanged, whereas TRPC6 mRNA expression levels showed 1.7- and 4-fold increases at the third and seventh day, respectively. These findings likely indicated that in UUO Piezo1 responded to mechanical stimuli, together with other TRP channels. Activation of Piezo1 in UUO may be largely attributed to tubular pressure, while in folic acid–induced kidney fibrosis, stretch and compression by deposited ECM, interstitial stiffness, and/or certain profibrotic factors (e.g., TGF-β_1_) may play important roles. Interestingly, inhibition of Piezo1 by GsMTx4 markedly attenuated UUO-or folic acid–induced fibrosis in mice, strongly indicating the role of Piezo1 in the development of kidney fibrosis.

It should be noted that GsMTx4 is a specific inhibitor of cationic mechanosensitive channels, meaning that GsMTx4 not only inhibits Piezo1 but also has other targets (for example, TRP ion family) (48, 49). The stretch-evoked increase in [Ca^2+^]_i_ concentration in urothelial cells was significantly attenuated by GsMTx4 to the level of Piezo1-knockdown cells (50), suggesting that GsMTx4 is to inhibit Piezo1 effectively. In order to verify the role of Piezo1, Piezo1-specific agonist Yoda1 (no effect on Piezo2 or TRP ion family) was also used in our animal study. Piezo1 stimulation by Yoda1 induced fibrotic alterations in the kidney of mice, supporting the involvement of Piezo1 in fibrosis. However, Yoda1 did not affect Piezo1 protein expression. The detailed molecular and biophysical mechanism is still unknown but is probably its direct interaction with Piezo1 or long-range membrane-delimited effects (through a change in membrane tension or curvature of the membrane) (51). Nevertheless, inhibition of Piezo1 by GsMTx4, at least, partially, contributes to attenuation of kidney fibrosis, and activation of Piezo1 was involved in the development of renal fibrosis.

### Activation of Piezo1 by mechanical (stretch, compression, rigidity) or chemical (TGF-β_1_ or Yoda1) stimulation induces profibrotic responses in tubular cells.

Mechanical stretch in tubular epithelial cells (and other types of cells) (26, 27) is a well-established model mimicking the pathogenic effects of tubular distention, which can provide a mechanism to stimulate an expression of fibrosis-related proteins (47, 52). In our study, stretch or compression markedly increased the protein expression of Piezo1 in HK2 cells, and stretch induced increased intracellular calcium levels. Cyclic stretch or compression on HK2 or mPTCs caused significant increases of fibronectin and α-SMA, which were almost completely abolished by Piezo1 inhibition or silence, indicating that Piezo1 activation is essential for initiation of an early response to mechanical stimulation in renal tubular epithelial cells. During progression of fibrosis, kidney stiffens due to the replacement of compliant cells with rigid matrix, which is further increased by cross-linking of these matrix fibrils (21). Stiffness may provide a favored mechanical microenvironment to activate Piezo1. HK2 cells or mPTCs cultured on PA hydrogels with various levels of stiffness exhibited increased mRNA and protein levels of Piezo1, again, in association with increased protein expression of fibronectin and α-SMA. Therefore, stiffness may enhance the mechanosensory and mechanotransduction capacity of tubular cells, leading to a profibrotic alteration in HK2 cells or mPTCs, which was prevented when inhibiting activation of Piezo1.

TGF-β_1_ has been long considered a key mediator of renal fibrosis, which promotes the accumulation of the ECM by enhanced synthesis of ECM proteins and by inhibiting their degradation (1, 32). Interestingly, Piezo1 inhibition by pretreatment with GsMTx4 or Piezo1 silence by siRNA dramatically prevented TGF-β_1_–induced increases in fibronectin and α-SMA, and a decrease in E-cadherin in HK2 cells and mPTCs, suggesting a critical role of Piezo1 in initiation and persistence of fibrosis. This conception was further supported by the finding that specific Piezo1 agonist Yoda1 stimulated expression of fibrosis-related protein in HK2 cells and mPTCs.

### Piezo1 activation induced profibrotic response through calcium/calpain/integrin pathway.

Mechanical signals propagate from the ECM and converge on cell surface adhesion receptors’ integrins, which connect intracellularly to the cytoskeleton within focal adhesion units (47). These stretch-activated cation channels are of fundamental importance in sensing and transducing external mechanical stresses (47) in a Ca^2+^-dependent manner. Piezo1 stimulation by either stretch or Yoda1 induced Ca^2+^ influx–dependent increases in the [Ca^2+^]_i_. Piezo1 evoking instantaneous influx of calcium in tubular epithelial cells is probably a direct and immediate mechanotransduction response, and, likely, the first step leading to various intracellular events that may be involved in development of fibrosis. Indeed, increases in [Ca^2+^]_i_ have been found to play important roles in the development of renal interstitial fibrosis (53).

**Figure 12:**
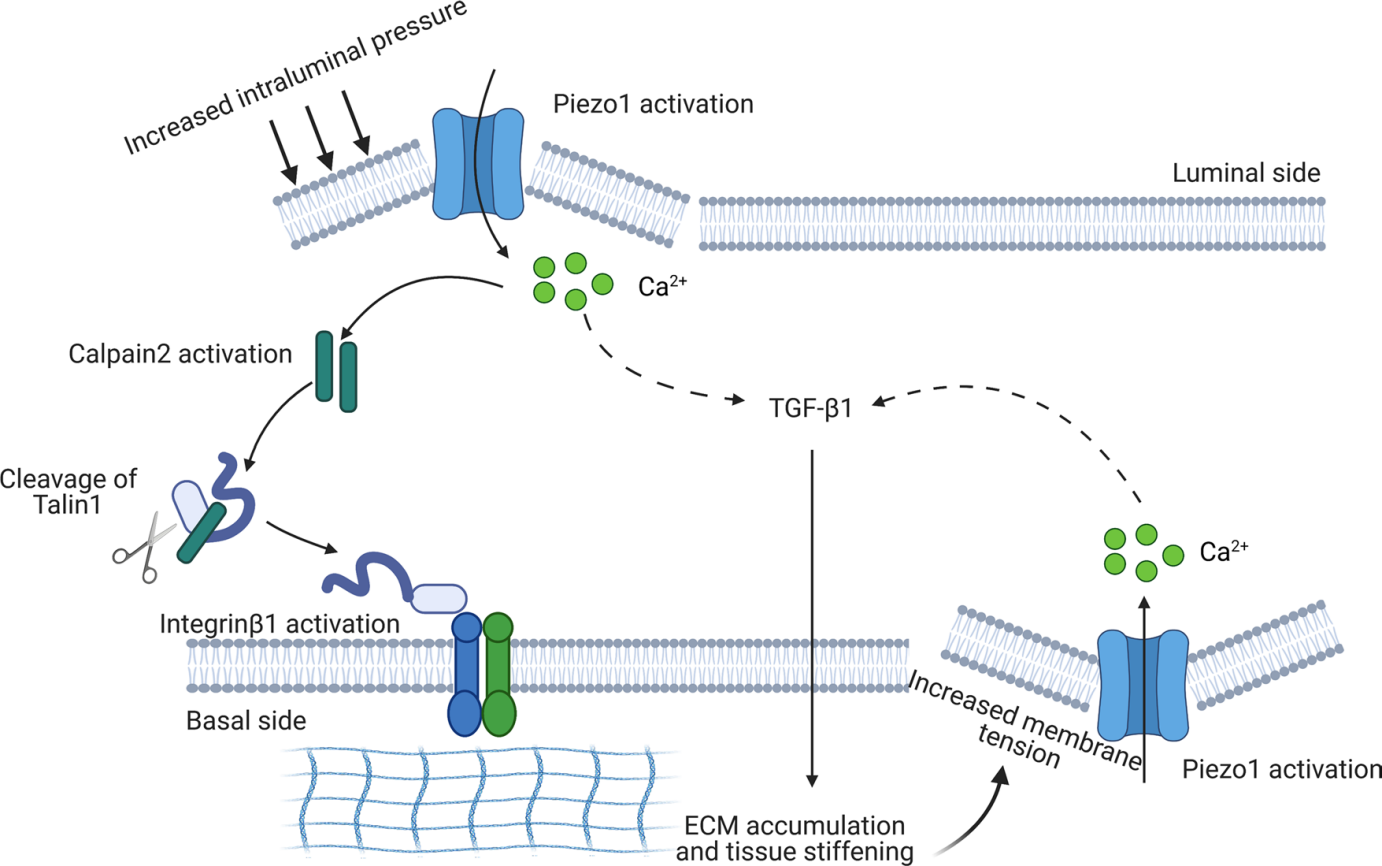
A potential reciprocal, Piezo1-dependent feed-forward mechanism in kidney fibrosis. During the progression of kidney fibrosis, the membrane tension of tubular epithelium (e.g., stretch or compression) is increased due to increased tissue stiffness induced by excessive deposition of ECM in tubulointerstitial areas and elevated intraluminal pressure, probably from fluid accumulation in tubules, leading to activation of Piezo1 and influx of calcium. By this way, mechanical signals are transduced into intracellular chemical signals. Intracellular calcium activates calpain2 that cleaves talin1 into active form, which subsequently causes activation and clustering of integrin β_1_ on the basolateral membrane, promoting the deposition of ECM (inside-out signal). Activation of Piezo1 also induces expression and synthesis of TGF-β_1_, further promoting ECM deposition. On the other hand, excessive deposition of ECM promotes tissue stiffening, which in turn increases the mechanosensory and mechanotransduction capacity of renal epithelial cells by activating Piezo1, aggravating the progression of renal fibrosis (outside-in signal).

Calpain system is integrated as a downstream signal of Piezo1 (30), and calpains are shown to play critical roles in adhesion disassembly and in focal adhesion turnover (54). Calpain cleaves talin1, which activates integrin β_1_ clustering on the membrane and induces phosphorylation of FAK, a critical step in inducing profibrotic phenotype changes in HK2 cells. Both in vivo (55) and in vitro (52) studies revealed an important role of integrin β_1_ in inducing tubulointerstitial fibrosis and tubular profibrotic injuries. In the current study, either silencing of Piezo1 with siRNA or calpain2 knockdown substantially attenuated Yoda1-induced profibrotic alterations and prevented cleavage of talin1 and upregulation of integrin β_1_ protein as well as phosphorylation of FAK in HK2 cells. Our data also showed that coexpression of integrin β_1_ and fibronectin protein was markedly strengthened by stimulation of Piezo1 with Yoda1, indicating a closed binding of integrin β_1_ and ECM once Piezo1 was activated. Therefore, our data suggested that Piezo1-mediated Ca^2+^ influx and corresponding activation of the calpain/talin1/integrin β_1_ pathway may be involved in profibrotic phenotype alterations in HK2 cells when Piezo1 was activated (Figure 12).

### A reciprocal, Piezo1-dependent feed-forward mechanism in kidney fibrosis.

The interplay between physical forces and biochemical signaling pathways may regulate kidney fibrosis initiation and progression. In the UUO and FAN animal models, early inhibition of Piezo1 by GsMTx4 revealed a marked prevention of kidney fibrosis, indicating a possibility that Piezo1 activation is involved in initiation of fibrosis. Our unpublished data obtained from a renal ischemia/reperfusion (I/R) injury model showed increased mRNA expression levels of *Piezo1* in 24-hour I/R injury and in I/R-induced chronic kidney disease rat models (unpublished data), supporting a role of Piezo1 in the early stage of renal injury. Irrespective of the initial causes, it could be speculated that multiple insults to the renal parenchyma may stimulate Piezo1 in tubular epithelia, which causes Ca^2+^ influx and corresponding activation of calpain/talin1/integrin β_1_ signaling and ECM accumulation (an inside-out signaling), initiating early events of fibrosis. During fibrosis progression, kidney tissue stiffening provides a favorable mechanical microenvironment to activate Piezo1, the activity of which in a calcium-dependent manner promotes the assembly of focal adhesions and activates integrin/FAK signaling (an outside-in signaling). Piezo1 signaling regulates the expression of genes and proteins involved in ECM remodeling, which can further modulate tissue stiffness. In turn, the stiffer environment upregulates Piezo1 expression to increase the mechanosensory and mechanotransduction capacity of renal epithelial cells. Interestingly, TGF-β_1_ promoted expression and activation of Piezo1, inducing profibrotic changes in both HK2 and mPTCs; on the other hand, Yoda1 stimulation was associated with increased expression of TGF-β_1_. These data likely indicated a reciprocal, Piezo1-dependent feed-forward mechanism between TGF-β_1_ and tubular mechanotransduction. Taken together, our data demonstrated a reciprocal, fibrosis-aggravating feed-forward circuit between Piezo1-dependent mechanotransduction and aberrant tissue mechanics in tubular epithelial cells (Figure 12).

Our findings provide molecular insights into the integration between mechanical stimulation and biochemical signaling pathways in development of kidney fibrosis. We found increased expression of Piezo1 in kidney fibrosis and amelioration of fibrosis by Piezo1 inhibition. Piezo1 stimulation by crucial mechanical cues, e.g., stretch, compression, and matrix rigidity, TGF-β_1_, or chemical agonist Yoda1 promoted profibrotic responses in HK2 cells and mPTCs, through intracellular Ca^2+^/calpain/talin signaling that regulates concerted activation of integrin-associated FAK. Our findings highlight the importance of external mechanical stress, mechanical sensors (Piezo1), and intracellular biological signaling pathways in the development of renal fibrosis, suggesting that blockade of the Piezo1 is probably a therapeutic option to delay the progress of kidney fibrosis.

## Methods

### Animal models.

Ten-week-old male C57BL/6J mice used in these experiments were obtained from GemPharmatech. All mice were housed in an animal facility with a 12-hour light/12-hour dark cycle, with water and chow ad libitum.

UUO surgery was performed as previously described (56). To investigate whether Piezo1 was activated in the obstructed kidney with increased intraluminal pressure, mice were randomly divided into 4 groups: sham group, UUO for 30 minutes group, UUO for 1 hour group, and UUO for 3 hours group. To investigate whether Piezo1 was involved in UUO-induced kidney fibrosis, mice were randomly divided into 6 groups: sham group (3 days), UUO for 3 days (3UUO) group, and 3UUO + GsMTx4 group; sham group (7 days), UUO for 7 day group (7UUO), and 7UUO + GsMTx4 group. Mice were subcutaneously injected in the back with 10 mg/kg GsMTx4 dissolved in double-distilled H_2_O (vehicle) every other day, starting on the first day after the surgery. The sham and UUO groups were given identical volumes of vehicle. The mice were sacrificed on day 3 and day 7 postsurgery, and the obstructed kidneys were harvested for analysis.

To investigate whether Piezo1 was involved in folic acid–induced kidney fibrosis, mice were randomly divided into 3 groups: control group, folic acid group, and folic acid + GsMTx4 group. In folic acid group, mice were treated with 250 mg/kg folic acid (MedChemExpress) dissolved in 0.3 M sodium bicarbonate (vehicle) via intraperitoneal injection for 7 days. In folic acid + GsMTx4 group, mice were subcutaneously injected in the back with 10 mg/kg GsMTx4 every other day, starting on the first day before folic acid treatment. Mice in control group received identical volumes of vehicle via intraperitoneal injection. The mice were sacrificed after 4 weeks and kidneys were harvested for analysis. See additional animal model information in Supplemental Methods.

### Human kidney autopsy samples.

Human kidney autopsy specimens were obtained from the Center of Medicolegal Expertise, Sun Yat-sen University. Kidney specimens from decedents, dying of car accidents and without pathological changes, were used as normal controls. Basic characteristics of decedents are listed in supplemental materials (Supplemental Table 1).

### Cell culture and treatment.

Human proximal tubular cells (HK2 cells) were obtained from ATCC and grown in DMEM/F12 (Corning) containing 10% FBS (Quacell Biotechnology) and 1% penicillin/streptomycin (Corning) and maintained at 37°C in 5% CO_2_ atmosphere. Mouse kidney proximal tubular cells (mPTCs) were prepared as previously described (57). Briefly, mPTCs were isolated from 10-week-old C57BL/6J mice and cultured in DMEM/F12 (Corning) containing 10% FBS (Quacell Biotechnology) at 37°C in 5% CO_2_ atmosphere.

The HK2 cells and mPTCs were seeded on 6-well plates (Thermo Fisher Scientific) at a density of 2 × 10^5^ cells/well for 24 hours and were serum-starved for 12 hours. The medium was changed to fresh serum-free DMEM/F12 before treatment. For morphological assessments, the cells were fixed with 4% paraformaldehyde and photographed using a microscope (Leica) connected to a digital camera with a macroconversion lens. For profibrotic response experiments, HK2 cells and mPTCs were treated with cyclic stretch for 24 hours or TGF-β_1_ (5 ng/mL, R&D Systems) for 48 hours, accompanied with either GsMTx4 (1 μM or 5 μM, TAIJIA biotech) or Piezo1 siRNA (RIBOBIO) pretreatments. Yoda1 (0.2 μM, MedChemExpress) was used to stimulate HK2 cells for 1 to 24 hours, with or without Piezo1 siRNA. A total of 1 μM Yoda1 was used to stimulate mPTCs for 24 hours. All the cells were then collected for the protein or RNA analyses.

### Histologic analysis and immunofluorescence.

For histology, kidney tissues were fixed with 4% paraformaldehyde for paraffin embedding. Tissue slices (4 μm) of kidney were prepared and stained via Masson’s trichrome, immunohistochemistry (IHC), and immunofluorescence. Paraffin-embedded kidney sections used for IHC studies were dewaxed, rehydrated, and incubated with Piezo1 (catalog 15939-1-AP, Proteintech) or collagen I (catalog BA0325, Boster) primary antibodies overnight at 4°C. The sections were subsequently incubated with goat anti–rabbit IgG (H+L) secondary antibody (catalog 31460, Thermo Fisher Scientific), treated with diaminobenzidine, and counterstained with hematoxylin, and images were captured by a digital scanning microscope (Leica DM2000). Masson’s trichrome staining was used to further evaluate renal fibrosis.

Immunofluorescence staining was implemented as described in a previous study (56). The frozen sections of kidney tissue (8 μm) or HK2 cells cultured on coverslips (20 mm) from different groups were rinsed with PBS (Corning), fixed with 4% paraformaldehyde, and incubated with primary antibodies against Piezo1 (15939-1-AP, Proteintech, USA), AQP1 (sc-25287, Santa Cruz Biotechnology), AQP2 (sc-515770, Santa Cruz Biotechnology), fibronectin (BA1772, Boster), or integrin β_1_ (sc-9970, Santa Cruz Biotechnology) overnight at 4°C. After the cells or sections were washed 3 times and incubated with secondary antibodies conjugated with Alexa Fluor 488 (donkey anti-mouse, catalog 1226927, Thermo Fisher Scientific) and Alexa Fluor 555 (donkey anti-rabbit, catalog 1189904, Thermo Fisher Scientific), nuclei were stained with DAPI (Thermo Fisher Scientific). The samples were imaged using a fluorescence microscope (Leica DMi8).

### Electrophysiology.

HK2 cells were recorded under the voltage-clamp mode at room temperature using an integrated patch amplifier (Sutter Instrument). HK2 cells were recorded in artificial cerebrospinal fluid composed of the following (in mM): 124 NaCl, 3 KCl, 1.25 NaH_2_PO_4_, 1 MgCl_2_, 2 CaCl_2_, 26 NaHCO_3_, and 10 dextrose, bubbled with 95%O_2_/5% CO_2_. Patch pipettes (2–4 MΩ) were filled with the internal solution composed of the following (in mM): 140 CsCl, 10 HEPES, 0.2 CaCl_2_, 5 MgATP, and 0.5 Na_3_GTP, pH 7.2–7.3. The osmolarity of the internal solution was 275–290 mOsm. Puff pressure was used for application of Yoda1. The tip diameter of puff micropipettes is about 2–5 μm, and puff pressure is between 1 and 5 psi: the horizontal distance from the tip of the puff micropipette to the recorded cell is about 20–40 μm, and the angle between the puff micropipette and cell surface is about 45°C. The tip of the puff micropipette is vertically 20–40 μm above the recorded cell (*z* axis). Cationic currents were recorded by puffing Yoda1 (10 μM) for 350 ms to the patched cells at holding potential of –90 (*n* = 22), –45 (*n* = 19), 0 (*n* = 23), 45 (*n* = 21), and 90 mV (*n* = 15). Only cells with series resistances < 20 MΩ and input resistances > 100 MΩ were included in the analysis. Cells were excluded if their input resistances changed >15% or if their series resistances changed >10% over the experiment. Recordings were filtered at 1 kHz and digitized at 10 kHz.

### Cell mechanical stretch stress model.

HK2 cells were seeded onto BioFlex culture plates (collagen I coated, Flexcell International Corporation) at a density of 2 × 10^5^ cells/well for 24 hours. After being serum-starved for 12 hours, culture medium was replaced with fresh serum-free DMEM/F12. The BioFlex culture plates were placed on vacuum-based loading docks of the Flexcell FX-5000T apparatus (Flexcell International Corporation) in the incubator and subjected to pulsatile mechanical stretch (20% of equibiaxial elongation) at a frequency of 0.3 Hz for 24 hours. Nonstretched cells (control group) were exposed to identical experimental conditions but without mechanical stretch.

### Three-dimensional cultures and application of mechanical compression.

The Flexcell-5000C Compression Plus System (Flexcell International Corporation) was used to apply mechanical compression to HK2 cells. Cells were cultured in 1.5 mg/mL type I collagen at a density of 2 × 10^5^ cells/well for 24 hours. Cells were subjected to mechanical compression under the condition of 15 mmHg at a frequency of 1 Hz for 24 hours (58), treated with or without 5 μM GsMTx4. Cells cultured under the same condition with no stress were used as control.

### Plasmid and siRNA transfection.

For plasmid transfection, CAPN2-KD plasmid was constructed. Briefly, knockdown of CAPN2 in HK2 cells was performed by CRISPR/Cas9-guided genome editing. Nucleotide single-guide RNA sequences: single-guide RNA 5′-CTTCCTGACGAATCGCGCCA-3′ (223759291–223759349 binding site) were designed using sgRNA CRISPR design tool online (http://crispr.mit.edu) and subcloned into the pSpCas9(BB)-2A-puro (PX459) plasmid (Addgene, catalog 62988). After subcloning, plasmids were purified and verified by sequencing. HK2 cells were seeded into 6-well plates at 30%–40% confluence and transfected with PX459 plasmid encoding a target-specific single-guide RNA using Lipofectamine 3000 (Invitrogen) according to the manufacturer’s instructions. Puromycin was then added to select the transfected cells after 24-hour transfection. Knockdown efficiency for CAPN2 was assessed by Western blot.

HK2 cells were transfected at 60% confluence in DMEM/F12 without FBS using Lipofectamine 3000 (Invitrogen) according to the manufacturer’s instructions. The final Piezo1 siRNA (RIBOBIO) concentration was 100 nM. Piezo1 suppression was confirmed by Western blot and quantitative PCR. The sequence of Piezo1 siRNA is 5′-GGCAGCGCATGAACTTTCT-3′.

### Western blotting and Co-IP studies.

HK2 cells or kidney samples were lysed in protein lysis buffer for 15 minutes on ice before protein was extracted. Western blotting was performed by electrophoresis and incubation with primary antibodies against fibronectin, α-SMA (ab5694, Abcam; BM0002, Boster; 14395-1-AP, Proteintech), collagen I (BA0325, Boster), calpain2 (11472-1-AP, Proteintech), E-cadherin (20874-1-AP, Proteintech), integrin β_1_ (12594-1-AP, Proteintech), Piezo1 (15939-1-AP, Proteintech; 28511-1-AP, Proteintech), Smad2/3 (5678s, Cell Signaling Technology), p-Smad2/3 (8828, Cell Signaling Technology), p-FAK Tyr397 (sc-81593, Santa Cruz Biotechnology), talin1 (14168-1-AP, Proteintech), GAPDH (60004-1-Ig, Proteintech), and β-actin (66009-1-Ig, Proteintech), followed by the addition of horseradish peroxidase–labeled secondary antibodies goat anti–rabbit IgG (H+L) (catalog 31460, Thermo Fisher Scientific) and goat anti–mouse IgG (H+L) (catalog 31430, Thermo Fisher Scientific). The blots were visualized in an ECL detection system (catalog FD8020, FDbio), and densitometric analysis was performed using AlphaEase software.

The samples subjected to IP assay (Thermo Fisher Scientific) were incubated with an anti–integrin β_1_ (sc-9970, Santa Cruz Biotechnology) antibody in IP buffer overnight at 4°C. Protein A-sepharose beads (from the IP assay kit) were added to the samples, which incubated for another 12 hours. The samples were then washed and resuspended, and Western blotting was performed as described previously (56).

### Quantitative real-time PCR.

Total RNA was extracted using TRIzol reagent (Invitrogen) and then reverse-transcribed with reverse transcriptase (AG-BIO). Resulting cDNAs were quantified by real-time PCR using SYBR green master mix (AG-BIO) on the StepOnePlus system (Applied Biosystems). The sequences of the primers used are listed in supplemental materials (Supplemental Tables 2 and 3).

### Measurement of intracellular calcium levels.

To determine the intracellular calcium level, cells were incubated with 10 nM Fluo-4-AM probe (Beyotime) for 30 minutes at 37°C before being subjected to flow cytometry analysis (Gallios, Beckman Coulter) or fluorescence microscope (Leica DMi8).

### PA hydrogel.

Cell culture on PA hydrogels of different stiffness was performed as described (33). The HK2 cells and mPTCs were seeded on PA hydrogels and grown in DMEM/F12 with 10% FBS and 1% penicillin/streptomycin at different stiffness conditions (4 kPa, 8 kPa, 20 kPa, and 35 kPa), cultured for 5 days (HK2 cells) or 7 days (mPTCs), and then collected for protein and mRNA analysis. To determine whether Piezo1 mediated higher matrix stiffness–induced profibrotic effects in HK2 cells and mPTCs, HK2 cells were cultured on PA hydrogels for 1 day and mPTCs for 2 days, followed by 5 μM GsMTx4 for another 4 days or 5 days. The proliferation of HK2 cells on PA hydrogels was detected by using an EdU labeling kit (RIBOBIO) according to the manufacturer’s recommendations. EdU assay was used to detect the cell proliferative activity. Fluorescence images were obtained by florescence microscopy (Leica). The proportion of EdU-staining positive cells to the total cells labeled by Hoechst indicated the proliferative rate.

### Statistics.

Results are presented as the means ± SEM. Data were analyzed by 1-way ANOVA and Student-Newman-Keuls tests for multiple comparisons. Statistical significance was accepted at the *P* < 0.05 level. The data analysis showed that the variance was homogeneous and accorded with normal distribution.

### Study approval.

All animal procedures were approved by the Animal Care and Use Committee of Sun Yat-sen University (Ethics Committee of ZSSOM on Laboratory Animal Care SYXK 2019-0209; Guangzhou, China). The study related to human kidney autopsy specimens was conducted in accordance with the Declaration of Helsinki, 2013, and approved by the Ethics Committee of Zhongshan School of Medicine, Sun Yat-sen University (No. 004, approved on March 5, 2019).

## Author contributions

XZ, YK, BL, and JX performed the experiments. XZ, CL, and WW designed the study; analyzed and interpreted the results; and wrote and edited the manuscript. XZ, YK, BL, and JX assisted with main experiments. YL, NZ, JL, BJ, JC, CL, and WW provided essential reagents and techniques for this study and reviewed the manuscript. XZ, YK, BL, JX, YL, NZ, JL, BJ, JC, CL, and WW approved the final version of manuscript. CL and WW conceived and supervised the study.

## Supplementary Material

Supplemental data

## Version 1 03/01/2022

In-Press Preview

## Version 2 04/08/2022

Electronic publication
