# Supplementary material for: Mechanosensitive Piezo1 channels mediate renal fibrosis
Source: JCI Insight. 2022 Apr 8;7(7):e152330. doi: 10.1172/jci.insight.152330 (PMC9057604; doi:10.1172/jci.insight.152330)
Supplement: Supplemental data [file jciinsight-7-152330-s102.pdf]

Supplementary data Ⅰ

Methods and Materials

Table 1. Basic characteristics of decedents

Identifier	Sex	Age	Cause of Death	Clinical Death Diagnosis	Diagnosis of kidney disease
1	M	21	Traffic accident		Clear glomerular contour clear with no obvious tubular type in the renal tubules. No obvious infiltration of inflammatory cells was observed in the stroma and vasodilation
2	M	78	MODS		Renal cyst (R)
3	M	46	Acute pericardial tamponade caused by ruptured aortic dissection aneurysm bleeding		Kidney stones (R&L), Renal cyst (L)
4	M	79	MODS	Nephrotic syndrome; Hypoproteinemia hypokalemia Urinary infection；	Diffuse glomerular atrophy and fibrosis; Renal cyst (L)
5	F	62	Rupture, hemorrhage and formation of right retroperitoneal small aneurysms	Diabetic nephropathy，Chronic Nephropathy Phase V Maintenance hemodialysis，Renal Hypertension，Renal anemia，Uremic encephalopathy；Renal cyst (L) with calculi	Glomerular fibrosis (mostly)

MODS, multiple organ dysfunction syndrome.

Table 2. Sequence of primers for mouse

**Target gene**	**Primer sequence for mouse**
Piezo1-F	5’-GGAAGAGGACTACCTTGGTG-3’
Piezo1-R	5’-GCTGACCTTGTCACTGAAGA-3’
TRPC1-F	5’-GTCGCACCTGTTATTTTAGCTGC-3’
TRPC1-R	5’- TGGGCAAAGACACATCCTGC-3’
TRPC6-F	5’- AGCCAGGACTATTTGCTGATGG-3’
TRPC6-R	5’- AACCTTCTTCCCTTCTCACGA-3’
TRPV4-F	5’-ATGGCAGATCCTGGTGATGG-3’
TRPV4-R	5’- GGAACTTCATACGCAGGTTTGG-3’
TGF-β1-F,	5’-CTCCCGTGGCTTCTAGTGC-3’
TGF-β1-R	5’-GCCTTAGTTTGGACAGGATCTG-3’
α-SMA-F	5’- CCCAACTGGGACCACATGG-3’
α-SMA-R	5’-TACATGCGGGGGACATTGAAG-3’
Fibronectin-F	5’- ATGTGGACCCCTCCTGATAGT-3’
Fibronectin-R	5’- GCCCAGTGATTTCAGCAAAGG-3’
GAPDH-F	5’-TGACCTCAACTACATGGTCTACA-3’
GAPDH-R	5’-CTTCCCATTCTCGGCCTTG-3’

TRPC, transient receptor potential channel; TRPV, transient receptor potential vanilloid; TGF-β1, transforming growth factor-β1; SMA, smooth muscle actin.

Table 3. Sequence of primers for human

Target gene	Primer sequence for human
TGF-β1-F	5’-GGCCAGATCCTGTCCAAGC-3’
TGF-β1-F	5’-GTGGGTTTCCACCATTAGCAC-3’
Piezo1-F	5’-TGAAGCGGGAGCTCTACAAC-3’
Piezo1-R	5’-TCTCGTTGGCATACTCCACA-3’
GAPDH-F	5’-GGAGCGAGATCCCTCCAAAAT-3’
GAPDH-R	5’-GGCTGTTGTCATACTTCTCATGG-3’

TGF-β1, transforming growth factor-β1.

Animal models

To investigate whether activation of Piezo1 was involved in renal fibrosis, mice were randomly divided into two groups: control group and Yoda1 group. In Yoda1 group, mice were treated with 2.6mg/kg Yoda1 (MedChemExpress, China) in 400μL (1.1% dimethylsulfoxide (DMSO), 2.7% ethanol, 96.2% PBS) via intraperitoneal injection for 14 days. Yoda1 was first dissolved in DMSO at a concentration of 30mM and then diluted to 10mM in ethanol. Mice in control group received identical volumes of vehicle via intraperitoneal injection. The mice were sacrificed after 14 days and kidneys were harvested for analysis.

Supplementary data Ⅱ

Figure S1. (A-D) Representative immunoblots and corresponding densitometry analysis of Piezo1 protein abundance in the kidney of Sham, 3UUO, 3UUO+GsMTx4, 7UUO and 7UUO+GsMTx4 mice. Data are shown as mean ± SEM (n=4 in each group). * $P<0.05$ when compared with sham mice by one-way ANOVA with Student-Newman-Keuls test. UUO, unilateral ureteral obstruction; 3UUO, UUO for 3 days; 7UUO, UUO for 7days.

Figure S2. (A) and (B) Representative immunoblots and corresponding densitometry analysis of Piezo1, fibronectin, TGF-β1 and integrinβ1 protein abundance in the kidney of CTL and Yoda1 mice. Data are shown as mean ± SEM (n=7 in CTL group and n=8 in Yoda1 group). * $P<0.05$ when compared with CTL by unpaired student's t test. TGF-β1, transforming growth factor-β1.

Figure S3. (A) mRNA level of Piezo1 in HK2 cells. Data are shown as mean ± SEM (n=6 in each group). **(B)** Fluo-4 AM was added to HK2 cells pretreated with 5μM GsMTx4 followed by cyclic stretch for 24 hours at 20% elongation, and the fluorescence signals of calcium ions in each group were detected by flow cytometry analysis. **(C)** and **(D)** Representative immunoblots and corresponding densitometry analysis of Piezo1 in HK2 cells transfected with Piezo1 siRNA. Data are shown as mean ± SEM (n=6 in each group). * $P<0.05$ when compared with scramble siRNA CTL by unpaired student's t test.

Figure S4. (A) and **(B)** Representative immunoblots and corresponding densitometry analysis of fibronectin protein abundance in HK2 cells cultured on polyacrylamide

hydrogels with 4KPa and 20KPa stiffness for 3 days, followed by treatment of TGF-β1 (5ng/ml) for another 2 days. Data are shown as mean ± SEM (n=4 in each group). * $P<0.05$ when compared with 4KPa CTL and # $P<0.05$ when compared with 20KPa CTL by one-way ANOVA with Student-Newman-Keuls test. TGF-β1, transforming growth factor-β1.

Figure S5. (A) and **(B)** Representative immunoblots and corresponding densitometry analysis of fibronectin and α-SMA protein abundance in HK2 cells cultured on polyacrylamide hydrogels with 4KPa and 20KPa stiffness, followed by treatment with GsMTx4. Data are shown as mean ± SEM (n=4 in each group). * $P<0.05$ when compared with 4KPa and # $P<0.05$ when compared with 20KPa by one-way ANOVA with Student-Newman-Keuls test. **(C)** and **(D)** Representative immunoblots and corresponding densitometry analysis of fibronectin and α-SMA protein abundance in mPTCs cultured on polyacrylamide hydrogels with 4KPa and 35KPa stiffness, followed by treatment with GsMTx4. Data are shown as mean ± SEM (n=4 in each group). * $P<0.05$ when compared with 4KPa and # $P<0.05$ when compared with 35KPa by one-way ANOVA with Student-Newman-Keuls test. mPTCs, mouse proximal tubular cells; SMA, smooth muscle actin.

Figure S6. (A) and **(B)** Representative immunoblots and corresponding densitometry analysis of integrinβ1 and p-FAK (Tyr397) protein abundance in the kidney of Sham, 7UUO and 7UUO+GsMTx4 mice. Data are shown as mean ± SEM (n=4 in each group). * $P<0.05$ when compared with Sham and # $P<0.05$ when compared with 7UUO mice

by one-way ANOVA with Student-Newman-Keuls test. 7UUO, unilateral ureteral obstruction for 7days; FAK, focal adhesion kinase.

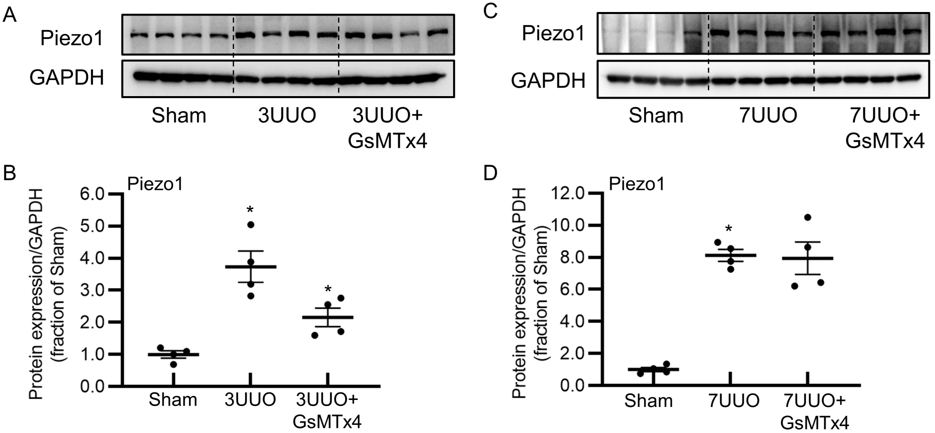

Figure S1. (A-D) Representative immunoblots and corresponding densitometry analysis of Piezo1 protein abundance in the kidney of Sham, 3UUO, 3UUO+GsMTx4, 7UUO and 7UUO+GsMTx4 mice. Data are shown as mean ± SEM (n=4 in each group). * $P<0.05$ when compared with sham mice by one-way ANOVA with Student-Newman-Keuls test. UUO, unilateral ureteral obstruction; 3UUO, UUO for 3 days; 7UUO, UUO for 7days.

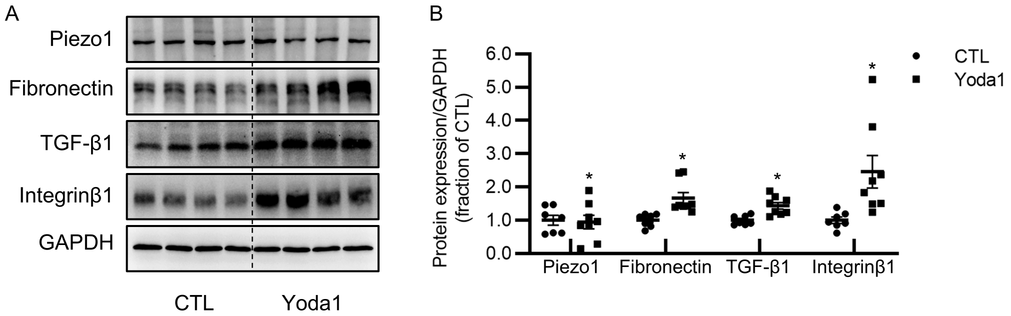

Figure S2. (A) and (B) Representative immunoblots and corresponding densitometry analysis of Piezo1, fibronectin, TGF-β1 and integrinβ1 protein abundance in the kidney of CTL and Yoda1 mice. Data are shown as mean ± SEM (n=7 in CTL group and n=8 in Yoda1 group). * $P<0.05$ when compared with CTL by unpaired student's t test. TGF-β1, transforming growth factor-β1.

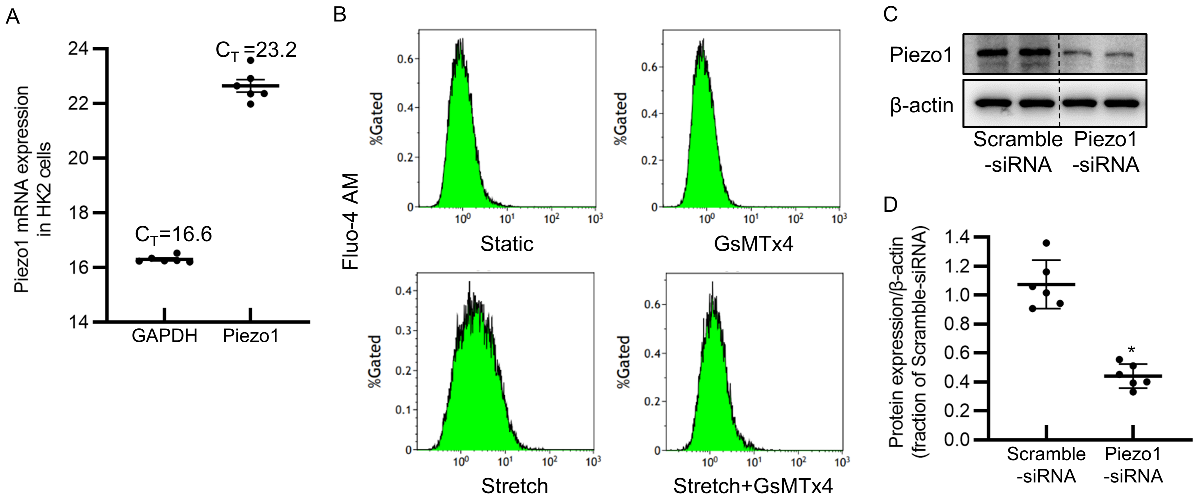

Figure S3. (A) mRNA level of Piezo1 in HK2 cells. Data are shown as mean ± SEM (n=6 in each group). **(B)** Fluo-4 AM was added to HK2 cells pretreated with 5μM GsMTx4 followed by cyclic stretch for 24 hours at 20% elongation, and the fluorescence signals of calcium ions in each group were detected by flow cytometry analysis. **(C)** and **(D)** Representative immunoblots and corresponding densitometry analysis of Piezo1 in HK2 cells transfected with Piezo1 siRNA. Data are shown as mean ± SEM (n=6 in each group). * $P<0.05$ when compared with scramble siRNA CTL by unpaired student's t test.

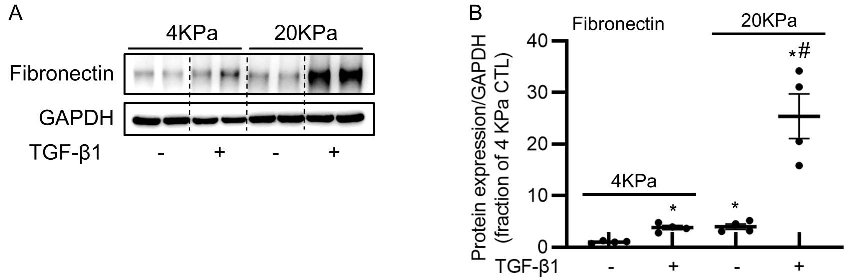

Figure S4. (A) and **(B)** Representative immunoblots and corresponding densitometry analysis of fibronectin protein abundance in HK2 cells cultured on polyacrylamide hydrogels with 4KPa and 20KPa stiffness for 3 days, followed by treatment of TGF-β1 (5ng/ml) for another 2 days. Data are shown as mean ± SEM (n=4 in each group). * P<0.05 when compared with 4KPa CTL and # P<0.05 when compared with 20KPa CTL by one-way ANOVA with Student-Newman-Keuls test. TGF-β1, transforming growth factor-β1.

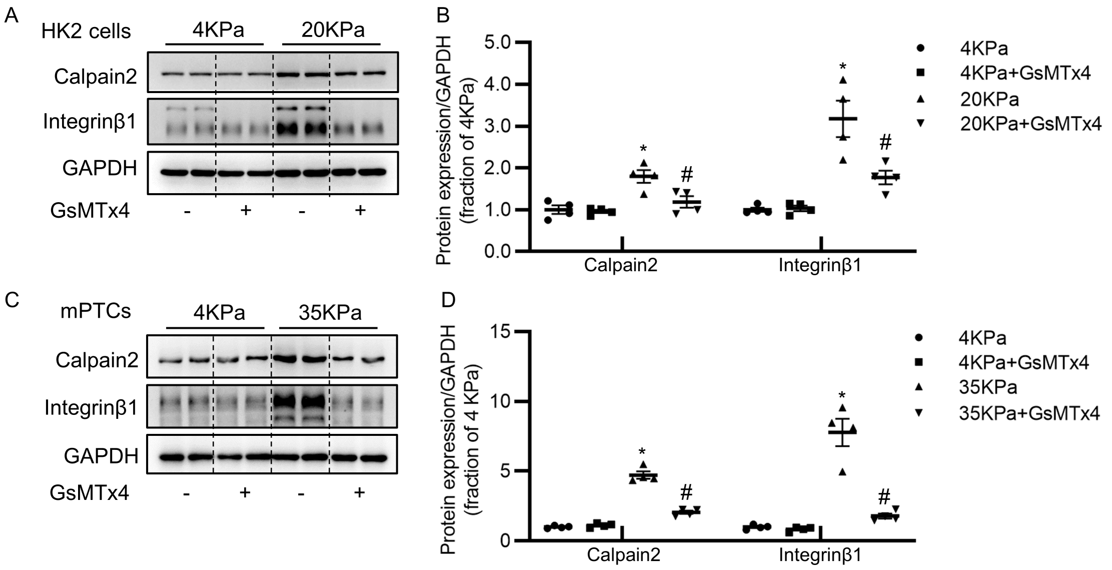

Figure S5. (A) and **(B)** Representative immunoblots and corresponding densitometry analysis of fibronectin and α-SMA protein abundance in HK2 cells cultured on polyacrylamide hydrogels with 4KPa and 20KPa stiffness, followed by treatment with GsMTx4. Data are shown as mean ± SEM (n=4 in each group). * P<0.05 when compared with 4KPa and # P<0.05 when compared with 20KPa by one-way ANOVA with Student-Newman-Keuls test. **(C)** and **(D)** Representative immunoblots and corresponding densitometry analysis of fibronectin and α-SMA protein abundance in mPTCs cultured on polyacrylamide hydrogels with 4KPa and 35KPa stiffness, followed by treatment with GsMTx4. Data are shown as mean ± SEM (n=4 in each group). * P<0.05 when compared with 4KPa and # P<0.05 when compared with 35KPa by one-way ANOVA with Student-Newman-Keuls test. mPTCs, mouse proximal tubular cells; SMA, smooth muscle actin.

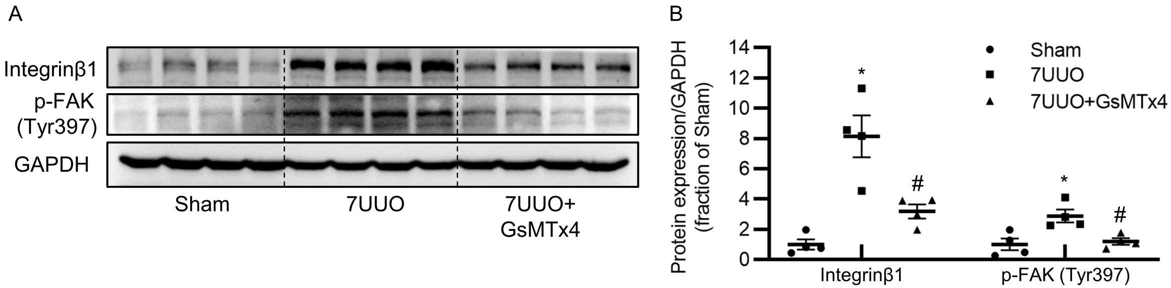

Figure S6. (A) and **(B)** Representative immunoblots and corresponding densitometry analysis of integrinβ1 and p-FAK (Tyr397) protein abundance in the kidney of Sham, 7UUO and 7UUO+GsMTx4 mice. Data are shown as mean ± SEM (n=4 in each group). * $P<0.05$ when compared with Sham and # $P<0.05$ when compared with 7UUO mice by one-way ANOVA with Student-Newman-Keuls test. 7UUO, unilateral ureteral obstruction for 7days; FAK, focal adhesion kinase.
